# NAC transcription factor family genes are differentially expressed in rice during infections with Rice dwarf virus, Rice black-streaked dwarf virus, Rice grassy stunt virus, Rice ragged stunt virus, and Rice transitory yellowing virus

**DOI:** 10.3389/fpls.2015.00676

**Published:** 2015-09-09

**Authors:** Mohammed Nuruzzaman, Akhter M. Sharoni, Kouji Satoh, Mohammad Rezaul Karim, Jennifer A. Harikrishna, Takumi Shimizu, Takahide Sasaya, Toshihiro Omura, Mohammad A. Haque, Sayed M. Z. Hasan, Aziz Ahmad, Shoshi Kikuchi

**Affiliations:** ^1^Plant Genome Research Unit, Agrogenomics Research Center, National Institute of Agrobiological SciencesTsukuba, Japan; ^2^Faculty of Science, Centre for Research for Biotechnology for Agriculture, Institute of Biological Sciences, University of MalayaKuala Lumpur, Malaysia; ^3^Post Harvest Technology, School of Food Science and Technology, University Malaysia TerengganuKuala Terengganu, Malaysia; ^4^Department of Agronomy and Agricultural Extension, Faculty of Agriculture, University of RajshahiRajshahi, Bangladesh; ^5^Research Team for Vector-Borne Plant Pathogens, National Agricultural Research CenterTsukuba, Japan; ^6^Centre for Fundamental and Liberal Education, School of Science and Food Technology, Universiti Malaysia TerengganuKuala Terengganu, Malaysia

**Keywords:** NAC transcription factors, virus infections, differential gene expression, *cis*-element, gene duplication

## Abstract

Expression levels of the *NAC* gene family were studied in rice infected with *Rice dwarf virus* (RDV), *Rice black-streaked dwarf virus* (RBSDV), *Rice grassy stunt virus* (RGSV), *Rice ragged stunt virus* (RRSV), and *Rice transitory yellowing virus* (RTYV). Microarray analysis showed that 75 (68%) *OsNAC* genes were differentially regulated during infection with RDV, RBSDV, RGSV, and RRSV compared with the control. The number of *OsNAC* genes up-regulated was highest during RGSV infection, while the lowest number was found during RTYV infection. These phenomena correlate with the severity of the syndromes induced by the virus infections. Most of the genes in the NAC subgroups NAC22, SND, ONAC2, ANAC34, and ONAC3 were down-regulated for all virus infections. These *OsNAC* genes might be related to the health stage maintenance of the host plants. Interestingly, most of the genes in the subgroups TIP and SNAC were more highly expressed during RBSDV and RGSV infections. These results suggested that *OsNAC* genes might be related to the responses induced by the virus infection. All of the genes assigned to the TIP subgroups were highly expressed during RGSV infection when compared with the control. For RDV infection, the number of activated genes was greatest during infection with the S-strain, followed by the D84-strain and the O-strain, with seven *OsNAC* genes up-regulated during infection by all three strains. The *Os12g03050* and *Os11g05614* genes showed higher expression during infection with four of the five viruses, and *Os11g03310, Os11g03370*, and *Os07g37920* genes showed high expression during at least three viral infections. We identified some duplicate genes that are classified as neofunctional and subfunctional according to their expression levels in different viral infections. A number of putative *cis*-elements were identified, which may help to clarify the function of these key genes in network pathways.

## Introduction

The *NAC* gene family name was derived from the names of three transcription factors: NAM (no apical meristem, Petunia), ATAF1–2 (*Arabidopsis thaliana* activating factor), and CUC2 (cup-shaped cotyledon, Arabidopsis), which share the same DNA-binding domain (Souer et al., 1996; Aida et al., 1997). *NAC* genes are found across a wide range of plant species and represent one of the largest families of plant-specific transcription factors. Over 50 families of different transcription factors have been identified in plants, based on sequence analyses of model species such as rice (Xiong et al., 2005). Together with *cis*-acting elements, transcription factors are involved in almost all aspects of cellular activity as part of their interrelated roles in regulating gene expression, as reviewed in Xiong et al., 2005). The availability of several complete plant genomic sequences, has led to the identification of 117 *NAC* genes in Arabidopsis, 151 in rice, 79 in grape, 26 in citrus, 163 in poplar, and 152 each in soybean and tobacco (Rushton et al., 2008; Hu et al., 2010; Nuruzzaman et al., 2010, 2012a; Le et al., 2011).

In recent years there has been improved knowledge on the molecular mechanisms underlying signaling pathways (Liu et al., 2010) and their involvement in activating defense responses in rice (Valent and Khang, 2010) and of rice innate immune responses, host recognition of pathogens (Skamnioti and Gurr, 2009) and recognition-triggered early signaling events. Microarray profiling after virus infection with *Rice stripe virus* in rice seedlings has disclosed 6 *OsNAC* genes induced via this virus infection (Nuruzzaman et al., 2010). Rice plants with a mutation in *rim1-1* are resistant to infection through the *Rice dwarf virus* (RDV) (Yoshii et al., 2009; Satoh et al., 2011). *NAC* genes have also been reported in viral infections in other crops, including the induction of the *StNAC* gene in response to *Phytophthora infestans* infection in the potato (Collinge and Boller, 2001); the expression of NAC proteins GRAB1 and GRAB2, which interact with the dwarf geminivirus RepA protein to control geminivirus DNA replication related with plant growth and development stages in wheat (Xie et al., 1999; Ren et al., 2000); and the expression of TaNAC4, which functions as a transcriptional activator required in the wheat response to both abiotic and biotic stresses (Xia et al., 2010). Several studies have elucidated the mechanisms that regulate the innate immune response in rice blast disease (i.e., OsNAC111) during infection by *Magnaporthe oryzae* (Yokotani et al., 2014), leading to the characterization of multiple disease resistance genes (*R* genes) (Liu et al., 2010). Lin et al. (2007) reported that an *OsNAC19* transcript was raised during infection with *Magnaporthe grisea*, advising that *OsNAC19* is needed in the rice defense mechanism to rice blast disease. A *NAC* gene (*NAM-B1*) plays an important role in nutrient remobilization from leaves to developing grains in wheat (Uauy et al., 2006). As seen in Arabidopsis, signaling pathways involving salicylic acid (SA) and ethylene (ET)/jasmonic acid (JA) are critical for the activation innate immunity in rice and cooperate using some common biochemical events (Chern et al., 2005; Qiu et al., 2007; Yuan et al., 2007; Li et al., 2011). Several regulatory proteins, including transcription factors such as OsNAC6, regulate defense responses against *Magnaporthe grisea* (Nakashima et al., 2007). However, a clear picture the molecular network regulating the rice immune response against pathogen/virus infection remains to be shown. The role of *NAC* genes in plant responses to abiotic stresses such as drought, salinity, cold, and submergence have also been widely reported (Hegedus et al., 2003; Hu et al., 2006, 2008; Jeong et al., 2010; Nuruzzaman et al., 2012b; Sun et al., 2015).

Genes in the NAC family have been shown to regulate different stages of plant life cycle such as rice flag leaves (Sperotto et al., 2009), embryo and shoot apical meristems in Arabidopsis (Duval et al., 2002), salt-responsive flowering in Arabidopsis (Kim et al., 2007a), xylem fiber development in Arabidopsis (Ko et al., 2007), lateral root development in Arabidopsis (Xie et al., 2000), and cell division (Kim et al., 2006). However, there is still a need for more characterization of the specific biological function of the various NAC family transcription factors, as these play important roles in the regulation of various processes related to plant physiology and responses to stress.

Previously, Nuruzzaman et al. (2010) showed a evolutionary relationship of NAC proteins based on the domain's structure and the subgroups of the phylogenetic tree fit well with according to the known NAC genes's function. Lin et al. (2007) exhibited that proteins with similar domains have the alike biological functions. Some well-known functions of NAC proteins are as follows: TIP NACs have been implicated in defense response and plant growth and development (Kim et al., 2007a,b, 2012; Yoshii et al., 2009); NAM/CUC3 is involved in shoot apical meristem formation and stress responses (Kim et al., 2007b; Nuruzzaman et al., 2012b, 2013); and SNAC group members are involved in responses to virus infection, abiotic stress and crosstalk between different signaling pathways (Nuruzzaman et al., 2012b, 2013). The SNAC subgroup includes known NAC members from rice, sugar cane (SsNAC23), Arabidopsis (such as ANAC72), and wheat (TtNAM-B1) that are involved in abiotic stress responses (Fang et al., 2008; Nuruzzaman et al., 2010, 2012b, 2013).

The NAC transcription factors modulate many target genes via the motif (such as CATGTG; Nakashima et al., 2007). In the promoter region of the selected gene and triggering transcription, this transcriptional regulatory system is called a regulon. Transcription factors belonging to specific gene family interact with the particular *cis*-elements, proteins, and their overexpression confers stress response in heterologous systems (Fujita et al., 2004; Tran et al., 2004; Hu et al., 2006).

The rice genome was predicted to contain 151 *NAC* genes (Nuruzzaman et al., 2010), and only a few of these genes have been characterized (Hu et al., 2006; Nakashima et al., 2007; Sperotto et al., 2009; Jeong et al., 2010; Takasaki et al., 2010; Sun et al., 2013; Yokotani et al., 2014). Originally this manuscript is the transcriptome analyses of rice *NAC* genes. In the case of RSV, RTSV, RDV, and RGSV infections, whole transcriptome analysis paper were already published (Satoh et al., 2011, 2013). But in the case of RBSDV, RRSV, and RTYV no transcriptome papers have been published and in this manuscript, we are trying to publish the data. In the case of RDV infection, Kimura et al. (1987) had isolated three different syndrome strains and in the whole transcriptome paper, we had compared the difference of transcriptome among three strains infection. From those data, *NAC* genes transcriptome data are extracted. And as for *NAC* gene transcriptome analysis, we have already published RSV infection data. This is why we have chosen five rice viruses and in the case of RDV infection, three strains data are given. Characterization of *NAC* family genes in rice can help us to understand the molecular mechanisms of resistance to stress, aiding in the development of rice varieties by using marker-assisted selection and transgenic technology. In this study, we investigated the expression of NAC family genes in seedlings and compared *OsNAC* gene expression during infection with three strains of *Rice dwarf virus* (RDV-D84, -O, and -S), *Rice black-streaked dwarf virus* (RBSDV), *Rice grassy stunt virus* (RGSV), *Rice ragged stunt virus* (RRSV), and *Rice transitory yellowing virus* (RTYV) on different days post-inoculation (dpi). To identify the putative virus-responsive genes in different rice plants, we used the Agilent 44K oligo array system to profile the transcriptomes of *OsNAC* genes during different virus infections, and we compared the different gene expression patterns. Several specific genes, or subgroups of this gene family, revealed novel information pertaining to their role in the plant response to virus infection. We also analyzed the segmental and tandem duplications of *OsNAC* genes to show conserved *cis*-elements in the 2-kb region upstream of the promoters of differentially expressed genes (DEGs) during virus infection. To our knowledge, this is the first report that focuses on the *OsNAC* genes to identify family-level expression patterns on different viruses. Taken together, these results provide a solid basis for future functional genomic research of *OsNAC* genes.

## Materials and methods

### Plant materials

Rice seeds (*Oryza sativa* L. ssp. *japonica* cv. “Nipponbare”) were supplied by Drs. M. Yano and T. Matsumoto of the National Institute of Agrobiological Sciences (NIAS), Japan.

### Inoculation of viruses

*Rice dwarf virus* (RDV; 3-strain RDV-D84, -O, and –S; Kimura et al., 1987), RBSDV, RGSV, RRSV, and RTYV were obtained from Dr. Toshihiro Omura, Research Team for Vector-Borne Plant Pathogens, National Agricultural Research Center, Tsukuba, Ibaraki, 305-8666, Japan. Experiments with RDV, RBSDV, RGSV, RRSV, and RTYV virus infections were performed at NIAS, Japan. RDV strains RDV-D84, -O, and -S were independently inoculated into rice seedlings via viruliferous green leafhopper (GLH: *Nephotettix cincticeps*). Inoculated plants were maintained in a temperature-controlled greenhouse (28 ± 3°C, natural sunlight). RDV-infected plants at 21 days post-inoculation (dpi); RBSDV-, RGSV-, and RRSV-infected plants at 28 dpi; and RTYV-infected plants at 24 dpi; at 21, 24, and 28 dpi, the shoots of the inoculated plants (excluding the meristem) were cut 3–5 cm above the soil surface. In short time we took the fresh weight, the plant samples were frozen in liquid nitrogen and stored at −80°C until extraction of RNA. This is a standard method, which has no affect the *NAC* gene expression.

### Estimation of virus accumulation

Shoots from 10 individual plants were collected at 0, 21, 24, and 28 dpi, as described above. Shoot extracts from the individual plants were diluted 1 in 10 (w/v) in phosphate-buffered saline and subjected to enzyme-linked immunosorbent assay (ELISA). The RDV accumulation in plants was estimated by ELISA as described by Shibata et al. (2007). We performed the same methods for the other viruses (RBSDV, RGSV, RRSV, and RTYV). All the experiments were repeated three times (biological replications).

### Microarray experiments

To reduce experimental variations, three sets of 10 seedlings were harvested from different viruses infected and mock-inoculated plants. Total RNA was prepared from the three replicate sets of the pooled samples (Table 1; biological replicates 1 through 3). To obtain a global picture of changes in gene expression in response to inoculation of rice plants with different viruses, three replicates were carried out in this study, using RNAs from biological replicate 1 as a technical replication. Extracting biological information from microarray data requires appropriate statistical methods. The simplest statistical method for detecting differential expression is the *t*-test, which can be used to compare two conditions when there is replication of samples. Cyanine-3 (Cy3)- and cyanine-5 (Cy5)-labeled target complementary RNA (cRNA) samples were prepared from 850 ng total mRNA using a fluorescent linear amplification kit (Agilent Technologies, USA) in accordance with the manufacturer's instructions. Transcriptome profiles specific to infected plants were examined by direct comparison of transcription activities between infected and uninfected plants on the same oligo array. Hybridization solution containing 825 ng of each of the Cy3- and Cy5-labeled cRNA preparations was prepared using an *in situ* Hybridization Kit Plus (Agilent Technologies, USA). The fragmented cRNAs were added to the hybridization buffer, applied to the microarray, and hybridized for 17 h at 60°C. The scanned microarray images were analyzed using Feature Extraction 6.1.1 software (Agilent Technologies, USA), and the dye-normalized, background-subtracted intensity and ratio data were exported to a text file. In cases where the software flagged corrupted spots or detected a lack of differences between sample spots and the background, these data were not included in further analysis. All arrays were performed in triplicate with independent samples.

**Table 1:** **Differentially expressed genes under different virus infections, >0.585 = Up-regulated and <0.585 = Down regulated**.

**Gene/Locus**	**Phylogenetic subgroup**	**RDV-84**	**RDV-O**	**RDV-S**	**RBSDV**	**RGSV**	**RRSV**	**RTYV**
*Os01g15640*	TIP	1.12	0.62	1.47		0.88		
*Os03g02800*	TIP/RIMI		−1.10	−2.03	0.78	1.14		
*Os05g35170*	TIP			0.70		0.86	0.82	
*Os06g01230*	TIP				0.89			
*Os08g44820*	TIP					1.14		
*Os09g38010*	TIP		−1.12	−3.48	2.09	1.57		
*Os10g42130*	TIP				3.57	5.44	2.14	
*Os01g01470*	NAM/CUC3					−1.75		
*Os01g29840*	NAM/CUC3							
*Os02g36880*	NAM/CUC3							
*Os03g21030*	NAM/CUC3	0.63						
*Os03g42630*	NAM/CUC3							
*Os04g38720*	NAM/CUC3				−1.02		−0.96	
*Os06g23650*	NAM/CUC3							
*Os07g48550*	NAM/CUC3					1.81		
*Os08g40030*	NAM/CUC3							
*Os11g03310*	NAM/CUC3	1.51	1.66	2.46	2.54	1.72		
*Os11g03370*	NAM/CUC3	1.89	2.08	2.25	1.53	1.48		
*Os12g03050*	NAM/CUC3	1.69	2.01	2.95	2.83	2.17	1.19	
*Os02g06950*	NAC1							
*Os04g52810*	NAC1							
*Os06g46270*	NAC1					1.23		
*Os08g10080*	NAC1				−3.04	−2.60		
*Os12g41680*	NAC1				1.93	1.70	1.02	
*Os02g41450*	NAC22				−3.08	−2.60	−1.20	
*Os02g56600*	NAC22				−0.68		−0.83	
*Os03g01870*	NAC22			2.05	3.48	3.29	1.99	0.71
*Os04g43560*	NAC22	0.86			−0.99			
*Os10g33760*	NAC22				−1.13		−0.86	
*Os02g15340*	SND							
*Os03g03540*	SND					2.68		
*Os04g59470*	SND						−1.16	−0.81
*Os06g01480*	SND		−1.36	−2.72				
*Os06g04090*	SND			−2.19				
*Os06g33940*	SND							
*Os08g01330*	SND				1.18	0.89		−1.97
*Os08g02300*	SND	−0.75	−0.67	−1.35				
*Os10g38834*	SND		0.70					
*Os01g66490*	ANAC34				−0.67	−0.69	−0.94	
*Os03g04070*	ANAC34					1.10	1.00	
*Os03g56580*	ANAC34			−0.72	−0.78			
*Os05g34600*	ANAC34							
*Os06g51070*	ANAC34			1.12		1.82	1.49	0.84
*Os07g04560*	ANAC34		−0.69	1.02	1.33	3.23	2.95	
*Os08g02160*	ANAC34							
*Os08g33910*	ANAC34					1.51		
*Os12g43530*	ANAC34						1.11	
*Os01g01430*	SNAC				−1.96	−1.15	−1.61	
*Os01g60020/OsNAC4*	SNAC	1.68				2.08	0.94	
*Os01g66120/SNAC2/6*	SNAC	1.20		1.11				
*Os02g12310/OsNAC3*	SNAC							
*Os03g21060*	SNAC					2.63	2.01	
*Os03g60080/SNAC1*	SNAC					1.35		
*Os05g34310*	SNAC							
*Os05g34830*	SNAC	1.05			1.08	0.61		
*Os07g12340*	SNAC					2.74		
*Os07g37920*	SNAC	2.15		2.89	3.15		1.91	
*Os07g48450*	SNAC				2.72		1.73	
*Os11g03300/OsNAC10*	SNAC				3.57	1.21	2.36	
*Os11g08210/OsNAC5*	SNAC					0.68		
*Os12g03040*	SNAC				3.27		2.33	
*Os01g09550*	ONAC4			−1.46				
*Os01g48130*	ONAC4			−1.04		−0.79		
*Os02g38130*	ONAC4		1.12	1.04	−1.36			
*Os04g40140*	ONAC4	1.46	1.79	2.18	0.73	1.35		
*Os05g10620*	ONAC4	1.63	1.57	2.17	0.70	1.76		
*Os05g48850*	ONAC4	−0.94	−0.91	−3.35		−1.63		
*Os06g15690*	ONAC4	1.49		1.69			0.59	
*Os10g25620*	ONAC4					−0.70		
*Os10g25640*	ONAC4							
*Os10g27360*	ONAC4							
*Os10g27390*	ONAC4							
*Os01g71790*	ONAC2	0.77					0.59	
*Os02g18470*	ONAC2							
*Os03g39050*	ONAC2							
*Os03g39100*	ONAC2							
*Os05g25960*	ONAC2	−0.68			−1.19		−1.63	
*Os07g09830*	ONAC2							
*Os07g09860*	ONAC2							
*Os07g17180*	ONAC2						−1.58	
*Os07g27330*	ONAC2							−0.97
*Os07g27340*	ONAC2							
*Os08g23880*	ONAC2							
*Os11g07700*	ONAC2							
*Os01g59640*	ONAC3				−1.49			
*Os03g59730*	ONAC3							
*Os07g31410*	ONAC3					−0.84		
*Os09g12380*	ONAC3							
*Os10g26240*	ONAC3							
*Os12g07790*	ONAC3	1.33			1.49			
*Os12g22630*	ONAC3							
*Os12g22940*	ONAC3				−2.05		−0.96	
*Os12g23090*	ONAC3							
*Os10g09820*	ONAC5							
*Os10g21560*	ONAC5	0.98		1.66				
*Os11g04960*	ONAC5							
*Os08g42400*	ONAC1					0.67		
*Os09g33490*	ONAC1							
*Os11g31330*	ONAC1							
*Os11g31340*	ONAC1							
*Os11g31360*	ONAC1						−1.36	
*Os12g29330*	ONAC1							
*Os01g64310*	ONAC7	2.91						
*Os05g37080*	ONAC7				1.39	3.11		
*Os11g05614*	ONAC7	1.72	1.48	2.13	1.29	3.85	1.62	
*Os11g45950*	ONAC7		1.98					
*Os12g05990*	ONAC7							
*Os02g34970*	ONAC6					3.14		
*Os04g35660*	ONAC6	−2.20						
*Os08g33670*	NEO	−1.09			−1.86		−1.96	
*Os09g24560*	NEO							
*Os09g32040*	OMNAC	0.68				0.68		

*Not differentially expressed genes are indicated as blank space. For convenience, the “LOC_” prefix has been omitted from the Osa1 locus IDs in the manuscript*.

*The OsNAC genes have been classified into 16 subgroups in rice (Nuruzzaman et al., 2010)*.

*RDV, Rice dwarf virus (RDV, three virus strains D84, -O, and-S); RBSDV, Rice black-streaked dwarf fijivirus; RGSV, Rice grassy stunt virus; RRSV, Rice ragged stunt orayzavirus; RTYV, Rice transitory yellowing virus*.

### Microarray-based gene expression data analysis

Gene expression data from all virus infections [*Rice dwarf virus* (RDV; 3-strain RDV-D84, -O, and -S; GSE24937), *Rice black-streaked dwarf virus* (RBSDV; GSE34263), *Rice grassy stunt virus* (RGSV; GSE25217), *Rice ragged stunt virus* (RRSV; GSE34265), and *Rice transitory yellowing virus* (RTYV; GSE34266)] are available at NCBI Gene Expression Omnibus GEO (platform number GPL7252) (http://www.ncbi.nlm.nih.gov/geo; or http://www.ncbi.nlm.nih.gov/gds; Barrett et al., 2009; or http://www.ncbi.nlm.nih.gov/gds/?term=GSE24937; http://www.ncbi.nlm.nih.gov/gds/?term=GSE34263; http://www.ncbi.nlm.nih.gov/gds/?term=GSE25217; http://www.ncbi.nlm.nih.gov/gds/?term=GSE34265; http://www.ncbi.nlm.nih.gov/gds/?term=GSE34266). The Cy3 and Cy5 signal intensities were normalized using rank-consistency filtering and the LOWESS method and processed by Feature Extraction version 9.5 (Agilent Technologies, USA). Expression patterns of all samples were transformed into log_2_–based numbers and normalized using EXPANDER version 5.0 (Shamir et al., 2005) according to the quantile method for the standardization of array slides. Differential expression (up- or down-regulation) was defined as a gene with a log_2_–based ratio (infected samples/control) ≥0.585 or ≤−0.585. A significant difference in gene expression profiles between the treated plants and the control was indicated by *P* ≤ 0.05 by paired *t*-test (permutations, all possible combinations; FDR collection, adjusted Bonferroni method). Data processing was performed with MeV version 4.4 (Saeed et al., 2006). We identified 112 *OsNAC* genes out of 151 (Nuruzzaman et al., 2010) from 44K microarray data collected during all virus infections.

### Cis-element analysis of the differentially expressed *OsNAC* genes

We performed *cis*-element analysis on the promoter sequences (2-kb upstream region) of specific differentially expressed *OsNAC* genes using Multiple Em for Motif Elicitation (MEME), a rice *cis*-element searching tool, and the Osiris site (RiCES; Doi et al., 2008; http://www.bioinformatics2.wsu.edu/cgi-bin/Osiris/cgi/home.pl).

### Gene duplications

The method of gene duplication analysis were published by Sharoni et al. (2011).

### Expression analysis by RT-PCR

RT-PCR was performed to confirm the differential expression of representative *OsNAC* genes identified by microarray data analysis using gene-specific primers. The complementary DNA (cDNA) fragments for transcripts of selected rice *NAC* genes or the selected virus (e.g., RDV) genome were synthesized using 1000 ng of the corresponding RNA with 50 ng/μl of random hexamer by SuperScript III reverse transcriptase (Invitrogen, USA). The resultant reaction mixtures containing cDNA were diluted four times. Four (4) μl of diluted mixture was used for PCR. Each PCR was performed in triplicate using an ABI 9700 Thermocycler (Applied Biosystems, USA) with incubation at 94°C for 1 min, 55°C for 50 s, and 72°C for 1 min. The RT-PCR runs consisted of 25–38 cycles, depending on the linear range of PCR amplification for each gene. For convenience, the “LOC_” prefix has been omitted from the Michigan State University (Osa1) locus IDs in this manuscript.

## Results

### Disease symptoms caused by different virus infections

**Figure 1:**
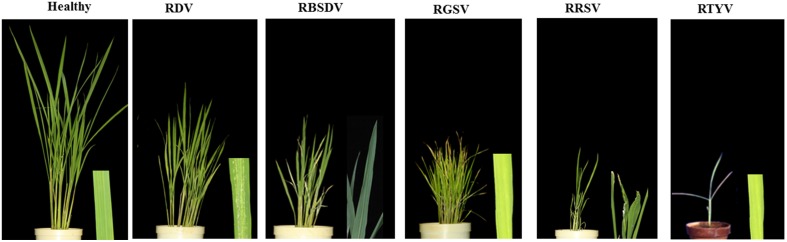
**Disease phenotype or virus accumulation in infected rice plants by different virus infections**.

In this study, we used five viruses mentioned in methods: RDV-infected plants at 21 dpi; RBSDV-, RGSV-, and RRSV-infected plants at 28 dpi; and RTYV-infected plants at 24 dpi, respectively (Figure 1). Rice plants infected with RDV showed disease symptoms such as stunted growth, chlorotic specks on leaves (Figure 1), and delayed and incomplete panicle exsertion (Hibino, 1996). Previously, heights of plants infected with three RDV strains at 40 dpi reported by Satoh et al. (2011). The rice plants infected with RBSDV, RGSV, RRSV, and RTYV were much shorter and leaf yellowing than healthy or mock/control (Figure 1). Profuse tillering became more evident in the RGSV-infected plants than mock reported by Satoh et al. (2013).

### *OsNAC* gene expression profiles with five virus infections

**Figure 2:**
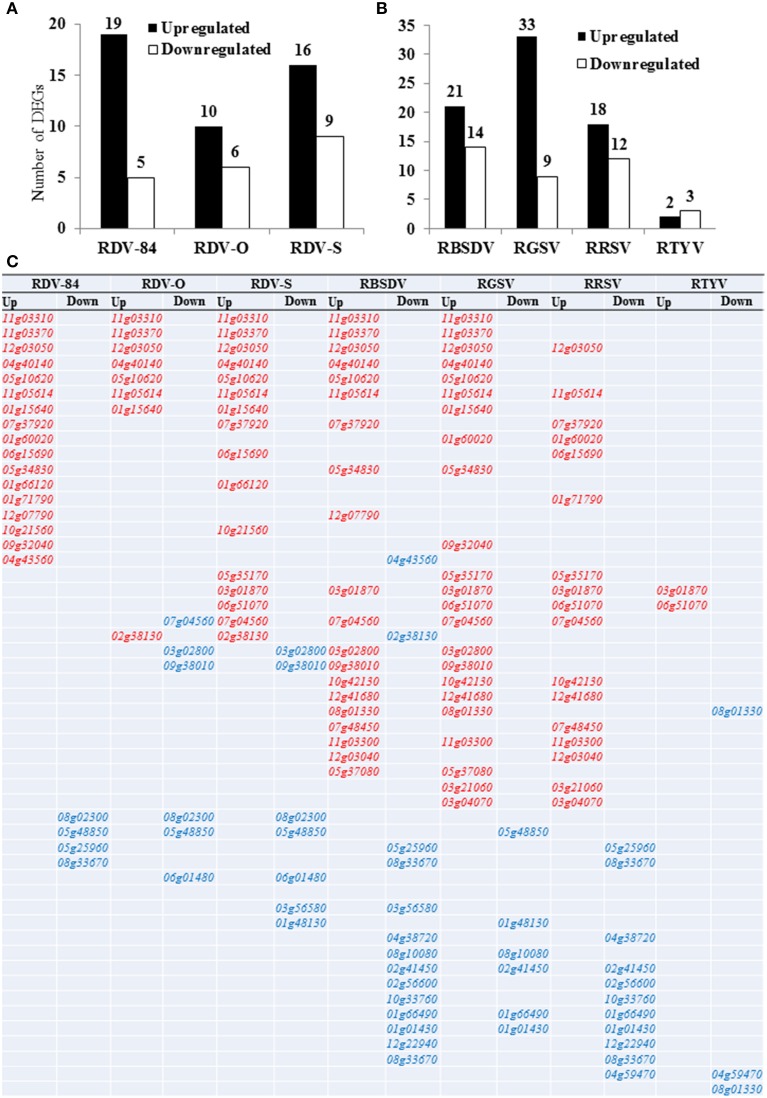
**Number of differentially expressed genes (DEGs) in rice seedlings (A) infected with RDV (3-strain) (B) infected with RBSDV, RGSV, RRSV, and RTYV respectively**. *Y*-axis represents the number of DEGs and different viruses are indicated on the *X*-axis. **(C)** Common or specific up- or down-regulated genes are mentioned among all the virus infections. Red color indicated the common up-regulated genes and blue color indicated the common down-regulated genes. These genes are specific up- or -down regulated to following virus infection: RDV-84, *01g64310, 03g21030*, and *04g35660*; RDV-O, *10g38834* and *11g45950*; RDV-S, *06g04090* and *01g09550*; RBSDV, *01g09550* and *01g59640*; RGSV, *08g44820, 07g48550, 06g46270, 03g03540, 08g33910, 03g60080, 07g12340, 11g08210, 08g42400, 02g34970, 01g01470, 10g25620*, and *07g31410*; RRSV, *12g43530, 07g17180*, and *11g31360*; RTYV, *07g27330*. Elaborations of virus infection are in Table 1.

To gain insight into the comprehensive roles of the *OsNAC* gene family members in response to various infections caused by viruses (RDV, RBSDV, RGSV, RRSV, and RTYV), their expression patterns were investigated in infected rice seedlings “(*Oryza sativa* L. ssp. *Japonica* cv. “Nipponbare”) by microarray analysis (Figure 2). Any selection based solely on fold change is arbitrary and there is no right nor wrong threshold. We compared the DEGs between 1.5 and 2 cutoff value under all virus infections and there is no gene up-regulated under RTYV infection (Table S1). We found small number of *NAC* genes in our array data, so we pick the threshold we feel is best for our experiment. We showed the data analysis and *P*-value of RDV-O and RTYV infections (for an example) in the Table S2. Only the genes whose expression change was at least 1.5-fold (increased or decreased) were considered to have responded to the above infections. Out of 151 *NAC* genes, we identified 112 *OsNAC* genes in our 44K array data. Among these genes, 75 were differentially expressed (up- or down-regulated) in at least one of the five virus infections (Figure 2; Table 1). The number of DEGs was different among plants infected with three RDV strains. The number of DEGs during infections with RDV-D84, RDV-O, and RDV-S was 24, 16, and 25, respectively (Figure 2). The number of genes up-regulated (33) was highest at 28 dpi during RGSV infection, followed by 21 and 24 dpi (listed in decreasing order) during RBSDV, RDV, RRSV, and RTYV infections (Figure 2). During infection with RDV, RBSDV, RGSV, and RRSV, there were higher numbers of up-regulated than down-regulated *OsNAC* genes. These up-regulated genes might be associated with the severity of the syndromes induced by the virus infections. However, in the case of RTYV infection, a higher number of *OsNAC* genes were down-regulated compared with the number up-regulated (Figure 2). Moreover, most of the genes in the NAC subgroups NAC22, SND, ONAC2, ANAC34, and ONAC3 were down-regulated on all days tested, during all virus infections (Table 1). These results indicated that *OsNAC* genes might be related to the health stage maintenance of the host plants. Interestingly, most of the genes in the subgroups TIP and SNAC were more highly expressed under RBSDV and RGSV infections.

We compared common and specific up- or down-regulated genes among all virus infections (Figure 2). Red color indicated the common up-regulated genes and blue color indicated the common donwregulated genes among the studied viruses. Specific up- or -down regulated to following virus infections were mentioned in the figure legend. Two genes *Os11g05614* and *Os12g03050* were common in all virus infections except RTYV infection (Figure 2C). Generally, among the strains of RDV tested, the degree of gene response to RDV-O infection was lowest and the degree of gene response to RDV-S infection was highest, with all infectious responses showing a greater number of up- than down-regulated *OsNAC* genes (Figures 2A,C). The *OsNAC* genes have been classified into 16 subgroups in rice (Nuruzzaman et al., 2010). We identified nine redundant *OsNAC* genes (7 up- and 2 down-regulated; top and down of the Figure 2C) that were common to rice plants infected by each of the three RDV strains (Figure 2C). Of the genes that seem to be differentially regulated in seedlings following infection by different strains of RDV, seven genes, *Os01g15640* (TIP), *Os11g03310, Os11g03370*, and *Os12g03050* (NAM/CUC3), *Os04g40140*, and *Os05g10620* (ONAC4), and *Os11g05614* (ONAC7), were commonly up-regulated across the plants infected by all strains (Figure 2; Table 1). Interestingly, the *Os04g40140* gene was also reported to be up-regulated under cold and GA treatments (Nuruzzaman et al., 2010). Table 1 shows that two genes (*Os01g66120/SNAC2/6* and *Os07g37920*) belonging to the SNAC (stress-responsive NAC) subgroup were up-regulated by RDV-84 and RDV-S infections. The role of SNAC during defense response is not clear yet, but SNAC may act as a positive regulator of defense mechanisms against pathogens because rice plants overexpressing one of the SNAC (*OsNAC6*) genes exhibited enhanced tolerance to *Magnaporthe grisea* (Nakashima et al., 2007). Comparatively speaking, the result of gene expression profile analysis for plants infected with five viruses suggested intercorrelation between the number of DEGs and the degree of gene responses.

### Choose the most promising putative candidate genes and subgroups during virus infection

To identify putative candidate genes that are responsible for virus infection responses in the seedlings, this study focused on genes that exhibited change expression in infected plants compared to control plants. The 10 genes expressed (up-regulated) during at least three virus infections are shown in Table 2. In the rice seedlings, eight genes, *Os10g42130* (TIP), *Os12g03050* (NAM/CUC3), *Os03g01870* (NAC22), *Os11g05614* (ONAC7), *Os07g37920, Os07g48450, Os11g0330*, and *Os12g03040* (SNAC), exhibited higher expression levels (≥2-fold) at 28 dpi during three virus infections (RBSDV, RGSV, and RRSV; Table 1). Four of these genes with higher expression levels during viral infection belonged to the SNAC subgroup. Six genes, including two genes mentioned above, (*Os11g03310, Os11g03370, Os12g03050, Os04g40140, Os05g10620*, and *Os11g05614* subgroups NAM/CUC3, ONAC4, and ONAC7, respectively) were highly activated (≥2-fold) in the seedlings infected with all RDV strains (RDV-84, -O, and -S), while two of them were activated during RBSDV, RGSV, and RRSV (Table 2). Interestingly, the *Os03g01870* (NAC22) gene was activated by RDV-S, RBSDV, RGSV, RRSV, and RTYV (Table 2). In addition, in the seedlings under different virus infection conditions, we noted that most of the genes assigned to the TIP and SNAC subgroups (86% and 50%, respectively) were highly expressed when compared with the control (Table 1). The results reported here suggest that up-regulation of the subgroup TIP and SNAC genes or specific candidate genes may be involved in regulating seedling development and in the response to different virus infections.

**Table 2:** **The 10 genes expressed (up-regulated) by at least three biotic treatments**.

**Gene name**	**P subgroup**	**RDV-84**	**RDV-O**	**RDV-S**	**RBSDV**	**RGSV**	**RRSV**	**RTYV**
*Os11g03310*	**NAM/CUC3**	1	1	1	1	1		
*Os11g03370*	**NAM/CUC3**	1	1	1	1	1		
*Os12g03050*	**NAM/CUC3**	1	1	1	1	1	1	
*Os03g01870*	NAC22			1	1	1	1	1
*Os07g04560*	ANAC34		1	1	1	1	1	
*Os07g37920*	**SNAC**	1		1	1		1	
*Os11g03300/OsNAC10*	**SNAC**				1	1	1	
*Os04g40140*	**ONAC4**	1	1	1	1	1		
*Os05g10620*	**ONAC4**	1	1	1	1	1		
*Os11g05614*	**ONAC7**	1	1	1	1	1	1	

*The OsNAC genes have been classified into 16 subgroups in rice (Nuruzzaman et al., 2010). Genes up-regulated more than 1.5 folds were assigned a value of 1. Genes of these bold subgroups are most important for further gene functional analysis*.

### Consensus *cis*-regulatory elements of selected genes

The *cis*-regulatory DNA sequences control gene responses in different tissues and constitute the essential functional linkage among gene regulatory networks. We determined that *cis*-motifs matching RNFG1, ABRE, GT-1, GCN4, GluB-1, BPBF, WRKY, TATAboxIII, ARF, and MYC were the most abundant *cis*-elements in the selected *OsNAC* genes that were differentially expressed during infection with one or more of the viruses (RDV, RBSDV, RGSV, and RRSV; Tables S3, S4). In the responses to virus infection, several elements (RNFG1OS, RYREPEATVFLEB4, ARFAT, PYRIMIDINEBOXHVEPB1, MYBGAHV, MYCATRD22, ACGTABREMOTIFA2OSEM, ABREOSRAB21, ACGTABREMOTIFA2OSEM, P-box, WBBOXPCWRKY1, BOXIINTPATPB, and GT1CORE) were linked to environmental stimuli such as ABA, GA3, SA, auxin, tissue-specific, light-harvesting, and drought. Most of the selected up-regulated *OsNAC* genes with tissue-specific expression profiles contained at least one of these *cis*-elements (Table S4), whereas uncommon *cis*-elements were found in the down-regulated *OsNAC* genes (data not shown).

### Comparison of expression profiles of duplicated *OsNAC* genes

In the course of evolution, there are three possible novel functions of gene duplication: nonfunctionalization, neofunctionalization, and subfunctionalization (Lynch and Conery, 2000). Divergence of gene expression plays a very important role in the preservation of duplicated genes. In this study, we examined the expression patterns of tandemly and segmentally duplicated genes during different virus infections. All the segmentally duplicated genes are in the same subgroups of *NAC* gene family, while some of the tandemly duplicates are not in the same subgroup. The tandem duplication gene structures in *NAC* gene subgroups *Os01g01430* (SNAC) and *Os01g01470* (NAM/CUC3) performed to be more variable and displayed the largest number of exon/intron structure variants compared with the other *NAC* genes. The dissimilarity of intron phases between subgroups and the conservation within *NAC* subgroups may mutually support to the results of phylogenetic analysis and genome duplication. We identified 14 clusters (18 pairs) of tandemly duplicated *OsNAC* genes (Table S5). Among them, only one cluster of the gene probe set was not found in our 44K microarray data. The expression patterns of seven clusters of tandemly duplicated genes (such as *Os03g21030* and *Os03g21060*) were dissimilar, which may indicate neofunctionalization (Figure S2A). Neofunctionalization occurs when a neofunctionalized allele is fixed in one of the duplicated genes and after duplication, one daughter gene retains the ancestral function while the other acquires new functions. The *Os03g21030* gene was down-regulated by RDV-O, RDV-S, RBSDV, RRSV, and RTYV, while the *Os03g21060* gene was up-regulated by all virus infections except RDV-84 (Figure S2A). Six clusters of genes (such as *Os11g03310* and *Os11g03370*) showed highly similar expression intensities, which may indicate subfunctionalization (Figure S2A). Likewise, we identified nine clusters (18 genes) of segmentally duplicated *OsNAC* genes (Table S6). All clusters of the gene probe set were found in our 44K microarray data. Four clusters of genes (such as *Os01g64310* and *Os05g37080*) showed highly dissimilar expression intensities, which may indicate neofunctionalization (Figure S2B). The expression patterns of five clusters of segmentally duplicated genes (such as *Os11g03300/SNAC10* and *Os12g03040*) were similar during all virus infections, which may indicate subfunctionalization (Figure S2B). On the basis of various roles, we predict that these duplicated genes may have diverse functions related to virus infections.

### Expression analysis by RT-PCR

**Figure 3:**
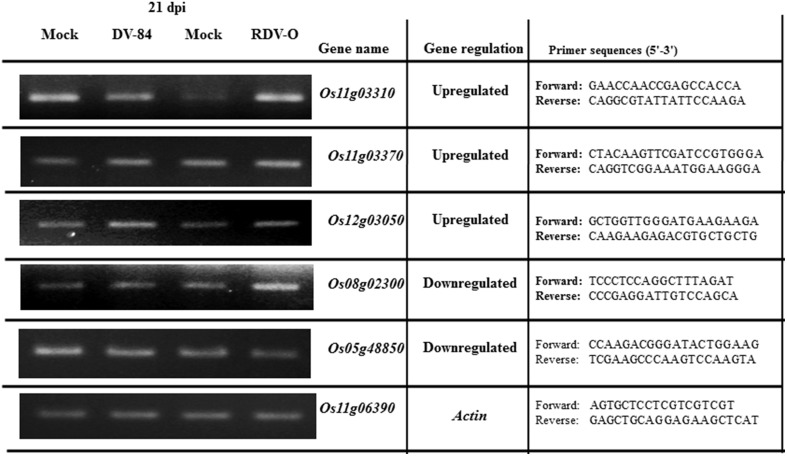
**Evaluation of the expression levels of selected DEGs by semi-quantitative RT-PCR during RDV-84 and RDV-O virus infections**.

To assess the accuracy of the microarray data, we selected five non-redundant genes that were differentially expressed in response to viral infection and examined the similarity between gene responses observed by microarray and by RT-PCR. Designed primers are shown in Figure 3. Rice *actin* gene (LOC_Os11g06390) was used for RT-PCR as an internal control, and its expression remained nearly constant under all experimental conditions (Figure 3). We observed that microarray and RT-PCR data, which were calculated based on the median of three repeated measurements, showed good correlation for RDV virus infection compared with the control; in most cases, the up-/down-regulated expression of five selected genes identified by microarray was also detected by RT-PCR (Figure 3). The oligoarray data of our lab have also been confirmed by Satoh et al. (2011, 2013). Hierarchical cluster analysis based on log_2_ ratio values showed that the *OsNAC* genes had very diverse expression profiles (red color for up and green color for down-regulation) during all virus infections (Figure S1).

## Discussion

During their life cycles, plants frequently encounter external stress conditions that adversely affect growth, crop yield, and development. Viruses are a major agricultural constraint; thus, understanding the responses of crops such as rice to virus infection is important for agricultural production. To establish infection in plants, viruses require host factors for their replication and for cell-to-cell and long-distance movement. To counterattack virus infection, plants have evolved different defense mechanisms, including up- or down-regulation of specific genes with various functions (Huang et al., 2010). *NAC* genes are key regulators of development and stress responses. The rice genome contains a predicted 151 *NAC* genes, of which most have not been characterized. Our goals in this study were to (i) determine the expression patterns of members of the *OsNAC* gene family in rice plants (Nipponbare) during infection by different viruses, (ii) assess the number of genes responding to different viruses, (iii) select the best candidate genes for further functional analysis, (iv) predict the important *cis*-elements in the promoter regions, and (v) determine the expression patterns of duplicated genes, which in turn may help to elucidate gene functions and gene networks. Several of the rice *NAC* gene family members showed a strong response to infection by one or more viruses in rice seedlings, confirming their association with responses to different virus infections. The Microarray is a test that allows for the comparison of thousands of genes at once. This test has cut away a great deal of time in experimentation, very little knowledge is available about many genes and just because mRNA is turned on doesn't mean proteins are made for that target gene(s). But it can make clue for further study to know the gene function(s).

### Diverse expression of the *OsNAC* gene family

Virus infection affects plant growth development and morphogenesis processes, and the disturbance of gene expression by virus infection may lead to the development of disease symptoms such as dwarfism and mosaic patterns on leaves (Shimizu et al., 2007). In the *OsNAC* gene family, 75 (68%) non-redundant genes were up-regulated during five tested virus infections (RDV, RBSDV, RGSV, RRSV, and RTYV; Figure 2; Table 1). The number of *OsNAC* genes with up-regulated expression was higher in infected compared with control plants (except for RTYV-infected plants), which indicates that defense systems were activated in RDV, RBSDV, RGSV and RRSV infections, while RTYV infection was nearly lethal (Figure 2). We observed that the number of genes up-regulated was highest at 28 dpi during RGSV infection, followed by 21 and 24 dpi (Figure 2). However, the multiplication of viruses may be inhibited by a plant's defense system during RGSV infection, suggesting that the defense system in the host was not activated quickly enough to suppress virus replication. One of the host defense systems against virus infection is the gene silencing system. The expression of genes involved in the gene silencing system is often activated by viral infection (Díaz-Pendón and Ding, 2008). Gene expression responses by plants infected with three RDV strains can be broadly categorized into three types: (i) responses that are similar in plants infected with RDV-D84 and RDV-O; (ii) responses that are similar in plants infected with RDV-D84 and RDV-S; and (iii) responses that are similar among all infected plants, independent of the RDV strain (Figure 2). The first response is mainly found in genes inactivated by RDV infection, where the degree of suppression in plants infected with RDV-D84 is lower than with RDV-O. The suppression of host gene expression in Nicotiana plants infected with the RNA virus *Cymbidium ringspot virus* showed that severe suppression of host genes was associated with the development of severe symptoms (Havelda and Várallyay, 2008). Therefore, the activation of genes for defense processes may be related to symptom development. Changes in gene expression in large numbers of *OsNAC* genes coincide with symptoms induced by infection of rice with the five different viruses tested in this study, leading us to suggest that members of this family contribute heavily to the plant response to virus infection. Among the *OsNAC* genes responding during virus infections, *Os11g03300/SNAC10* was expressed preferentially in the leaf during RBSDV, RGV, and RRSV; therefore, it is possible that these genes are involved in the regulation of rice seedling growth (Figure 2; Table 1). Interestingly, two genes, *Os12g03050* and *Os11g05614*, showed preferential expression during four virus infections, whereas three genes, *Os11g03310, Os11g03370*, and *Os07g37920*, showed preferential expression during three virus infections (Table 2). Interestingly, the *Os03g60080/SNAC1* gene is activated only by RGSV and is not differentially expressed by plants infected with the other viruses tested in our study (Table 1). We found that this is a key gene and it might be function under biotic and abiotic stress conditions (Hu et al., 2006). Satoh et al. (2013) reported that the gene expression profile of plants infected with RGSV suggests that symptoms such as stunting and leaf chlorosis are associated with the suppression of genes related to cell wall, hormone, and chlorophyll synthesis, while the excess tillering symptom specific to RGSV infection is associated with the suppression of strigolactone signaling and gibberellic acid metabolism. We identified 10 genes that were up-regulated in rice seedlings by at least three virus infections as strongly related to plant responses to viral infection (Table 2). The expressions of many *NAC* genes were changed by RDV infection. Notably, some genes in the SNAC (Takasaki et al., 2010) family were induced by RDV infection. Thus, as shown by suppression of transcription factor genes such as those encoding the homeobox-like HD-Zip genes, the responses of *NAC* genes seem to be dependent on the encoded domain types, which may be related to distinctive gene functions (Elhiti and Stasolla, 2009). These candidate defense genes, which are preferentially expressed in seedlings of different rice cultivars, may deserve special attention in further functional investigations.

### Function of different subgroups

*OsNAC* genes play crucial roles in various developmental processes, including signaling, stress responses, and plant defenses. We found that there is functional redundancy among the subgroups of the *OsNAC* gene family. In total, 11 (79%) *SNAC* genes were up-regulated during at least one of the five virus infections in this study (Table 1), which may indicate that *SNAC* genes can be activated via infection by different viruses. From our array analysis, we observed that rice *NAC* genes that were up-regulated by virus infection include those previously reported to be induced by abiotic stress (Nuruzzaman et al., 2010, 2012b). The subgroup NAM/CUC3 includes the known NAC protein CUC3, and other members in that subgroup also have homology with Arabidopsis NAM/CUC3 proteins. Five (42%) genes in this subgroup were induced by virus infection. Our microarray experiments showed that three genes (i.e., *Os11g03310, Os11g03370*, and *Os12g03050*) of the NAM/CUC3 subgroup were induced by infection with RDV (3 strains), RBSDV, and RGSV (Table 1). In our study, eight genes, *Os10g42130* (TIP), *Os12g03050* (NAM/CUC3), *Os03g01870* (NAC22), *Os07g37920* (SNAC), *Os07g48450* (SNAC), *Os11g0330* (SNAC), *Os12g03040* (SNAC), and *Os11g05614* (ONAC7), in this subgroup showed upregulation during different virus infections (RBSDV, RGSV, and RRSV; Table 1). There is high homology with known genes and tight clustering of members in each subgroup reported by Nuruzzaman et al. (2010). Although phylogenetic analysis provides important support for candidate gene selection, it alone cannot precisely indicate gene function. For this reason, our tissue expression analysis at the level of mRNA transcription and the conserved group-specific residues of the NAC domain defined in this study were used to select strong candidates for future investigation to further understand the function and relationships of NAC transcription factors.

### Responses of *OsNAC* genes to various treatment conditions

In this study, several *OsNAC* genes exhibited high or low expression during infection by different viruses (Figure 2; Table 1). We found that several of these virus-responsive *OsNAC* genes were among those we previously reported to be activated by at least one of the treatments with NAA, GA3, SA, ABA or JA, or abiotic treatments (cold, drought, and submergence) of rice seedlings (Nuruzzaman et al., 2010, 2012b). A number of plant *NAC* genes (e.g., AtNAC2) are affected by auxin, ethylene (Xie et al., 2000; He et al., 2005), and ABA (e.g., *OsNAC5*; Sperotto et al., 2009). In Arabidopsis, NAC transcription factor NTL8 regulates GA3-mediated salt signaling in seed germination (Kim et al., 2008). In this study, the *Os05g34830/SNAC* gene was induced specifically in the seedlings during infections with RDV-84, RBSDV, and RGSV (Figure 2; Table 1), and this same gene is also activated by ABA treatment (Nuruzzaman et al., 2012b). Therefore, this gene might be involved in the defense system during virus infections. The *OsNAC5, ONAC009, ONAC071*, and *OsNAC6* genes are homologs that are induced by abiotic stresses such as drought, high salinity, and ABA (Takasaki et al., 2010). Previously, we reported that the *Os03g21030, Os05g34830*, and *Os07g48550* genes were induced in the root, leaf, and panicle under both drought conditions and ABA treatments (Nuruzzaman et al., 2012b), and we observed these same genes responding to infections by RDV-84, RBSDV, and RGSV in the current study. From these results, we speculate that there are *OsNAC* genes that function in ABA signaling pathways that are involved in the defensive response against virus infections. The *Os03g60080/SNAC1* gene is activated by RGSV infection (Table 1) and is also up-regulated following treatment with GA3 and SA in rice seedlings (Nuruzzaman et al., 2012b). Similarly, the expression intensity of this gene is much greater in seedlings infected with RDV. However, *Os01g66120/SNAC2/6* (SNAC) is up-regulated by NAA, GA3, and KT (Nuruzzaman et al., 2012b), and this gene is also observed to respond to infections by RDV-84 and RVD-S in our study. Many of these genes that have similar expression profiles in rice after infection with different viruses function to encode signaling components, including transcription factors and protein kinases (Seki et al., 2002), as observed here with genes activated during virus infection that have been previously reported to be associated with various environmental and abiotic stress responses. Therefore, we infer that *OsNAC* genes might play functional roles in rice seedlings during different virus infections, and that these roles include signal transduction via growth factor pathways.

### The defense mechanism of *OsNAC* genes in rice

Transcription factors and *cis*-elements function in the promoter region of various stress-related genes, and overexpression of these genes may improve the plant's response to stress. We predicted that a number of gene-specific *cis*-elements might be important in the regulation of target genes by other factors, and that these *cis*-elements may influence the up-regulated genes during virus infection of the resistant rice plant (Tables S4, S5). We found that the expression levels in our RT-PCR results were very similar to the intensities of the microarray data, (Figure 3; Figure S1). Data on such expression patterns have been published for genes encoding proteins that contain protein kinase, leucine-rich, NB-ARC, and EF-hand domains, which might function in signal transduction for defense systems (Tameling and Baulcombe, 2007; Li et al., 2009). Host defense systems have been associated with genes for transcription factors in the WRKY family (*OsWRKY45*; Shimono et al., 2007). In this study, we found that several *OsNAC* genes, e.g., *Os11g03300/SNAC10* and *Os12g03040*; *Os11g03370* and *Os12g03050* belonging to the SNAC and NAM/CUC3 respectively, involved in the ABA, GA3, SA, auxin, tissue-specific, and light-harvesting responses were members of the SNAC and NAM/CUC3 subgroups and contained *cis*-elements (e.g., RNFG1OS) or *cis*-motifs (e.g., ABRE) in their upstream regions. Moreover, their expression may be induced by different virus infections (Tables S4, S5). It was shown that stress-inducible promoters such as the OsNAC6 and SNAC1 promoters are more suitable for overexpression to minimize negative effects on plant growth in transgenic rice (Hu et al., 2006; Nakashima et al., 2007). Some reports suggested that CaNAC1, BnNAC, and OsNAC6 members of the subgroup SNAC share common functions in the plant induction response to pathogen/virus infection (Table S7) and abiotic stresses (Oh et al., 2005; Nakashima et al., 2007). Thus, it is important to identify target genes for transcription factors involved in stress responses and to do comparative analysis of gene expression profiles during different virus infections to determine the functional role of *OsNAC* genes in the growth of the plant and its response to virus infection. With the help of bioinformational analysis, we predict that further analysis of a number of the above transcription factors will contribute to a deeper understanding of gene regulation in rice during different virus infections.

### Duplication and putative function of *NAC* genes

Coordinated patterns of gene regulation in plants may evolve through one or more of the processes of gene duplication, nucleotide substitution, domain duplication, and intron/exon shuffling. Among these, gene duplication is a major evolutionary mechanism leading to speciation and to functional diversification within plant genomes (Moore and Purugganan, 2003). In the *MYB* gene family, segmental duplications appear to have occurred relatively frequently, often involving tandem duplications in local genomic clusters with low levels of retention of segmental duplications such as in the disease-resistance NBS-LRR gene family (Cannon et al., 2004). We identified some duplicate genes, for example *Os03g21030, Os03g21060, Os11 g03300/SNAC10*, and *Os12g03040* (Figure S2), that are classified as neofunctional and subfunctional according to their expression level in different virus infections. Thus, we assume that there is functional redundancy among the *OsNAC* genes.

In conclusion, the application of a comprehensive 44K oligo array platform with unlike rice genotypes enabled us to define gene expression levels during the infection of rice seedlings with different viruses. By comparing the gene expression profiles for *NAC* family genes from all genotypes under virus-infected and -uninfected conditions, we identified several NAC family transcription factors that may be responsible for the virus response in rice seedlings. Together with the putative *cis*-elements identified in this study, the *NAC* family genes could be used as novel reference genes, toward clarifying their functions and pathways. This could be especially useful for determining the functions of the *OsNAC* family of genes in rice at the seedling stage of growth. The genes belonging to the TIP, SNAC, and NAM/CUC3 subgroups were activated in the seedlings and provide a new avenue for determining the best candidate genes for functional analysis. Some subgroups showed a high level of expression in virus infection, suggesting that they might have undergone functional divergence. Current work to characterize a number of these genes through overexpression and knockdown/mutant analyses is underway in our laboratory toward the optimization of molecular breeding schemes for the *OsNAC* gene family in rice.

## Author contributions

MN and SK designed the experiments. MN analyzed the data and assisted in writing the manuscript. AS and KS contributed materials and analysis tools. KS, MK, AH, AA, SH, and JH assisted in analyzing the data and writing the manuscript. All authors read and approved the final manuscript and agree to be accountable for all aspects of the work.

### Conflict of interest statement

The authors declare that the research was conducted in the absence of any commercial or financial relationships that could be construed as a potential conflict of interest.

## Supplementary material

Figure S1 **Differential expression of ***OsNAC*** genes under five types of biotic stresses (log_2_ ratio); Color bar at top shows level of expression**. Red indicates expressed genes and green indicates unexpressed ones; RDV, Rice dwarf virus (three virus strains D84, O, and S); RBSDV, *Rice black-streaked dwarf fijivirus*; RGSV, *Rice grassy stunt tenuivirus*; RRSV, *Rice ragged stunt virus*; and RTYV, *Rice transitory yellowing nucleorhabdo virus*.

Figure S2 **Examples of expression patterns of ***OsNAC*** genes found in duplicated regions of the rice genome. (A)** Expression patterns of two pairs of tandemly duplicated OsNAC genes. **(B)** Expression patterns of two *OsNAC* genes found in segmentally duplicated regions. The different biotic stresses are shown on the *X*-axis and the expression intensity is on the *Y*-axis. Gene names are indicated at the top of each graph. Elaborations of virus infection are in Table 1.
