# Supplementary figures and images for: NAC transcription factor family genes are differentially expressed in rice during infections with Rice dwarf virus, Rice black-streaked dwarf virus, Rice grassy stunt virus, Rice ragged stunt virus, and Rice transitory yellowing virus
Source: Front Plant Sci. 2015 Sep 9;6:676. doi: 10.3389/fpls.2015.00676 (PMC4563162; doi:10.3389/fpls.2015.00676)

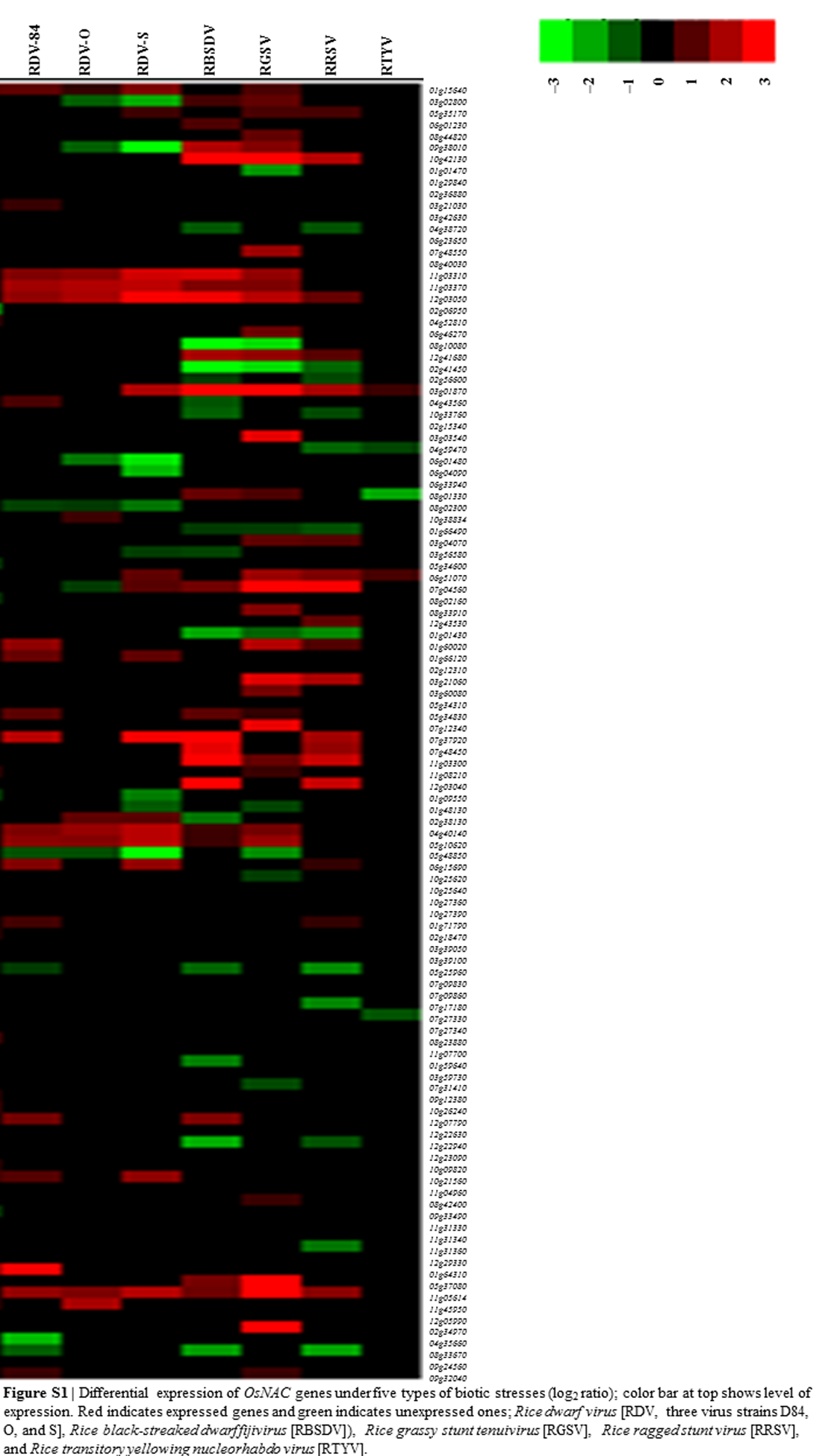

Supplement: Figure S1 — Differential expression of OsNAC genes under five types of biotic stresses (log2 ratio); Color bar at top shows level of expression. Red indicates expressed genes and green indicates unexpressed ones; RDV, Rice dwarf virus (three virus strains D84, O, and S); RBSDV, Rice black-streaked dwarf fijivirus; RGSV, Rice grassy stunt tenuivirus; RRSV, Rice ragged stunt virus; and RTYV, Rice transitory yellowing nucleorhabdo virus. [file Image1.TIF]

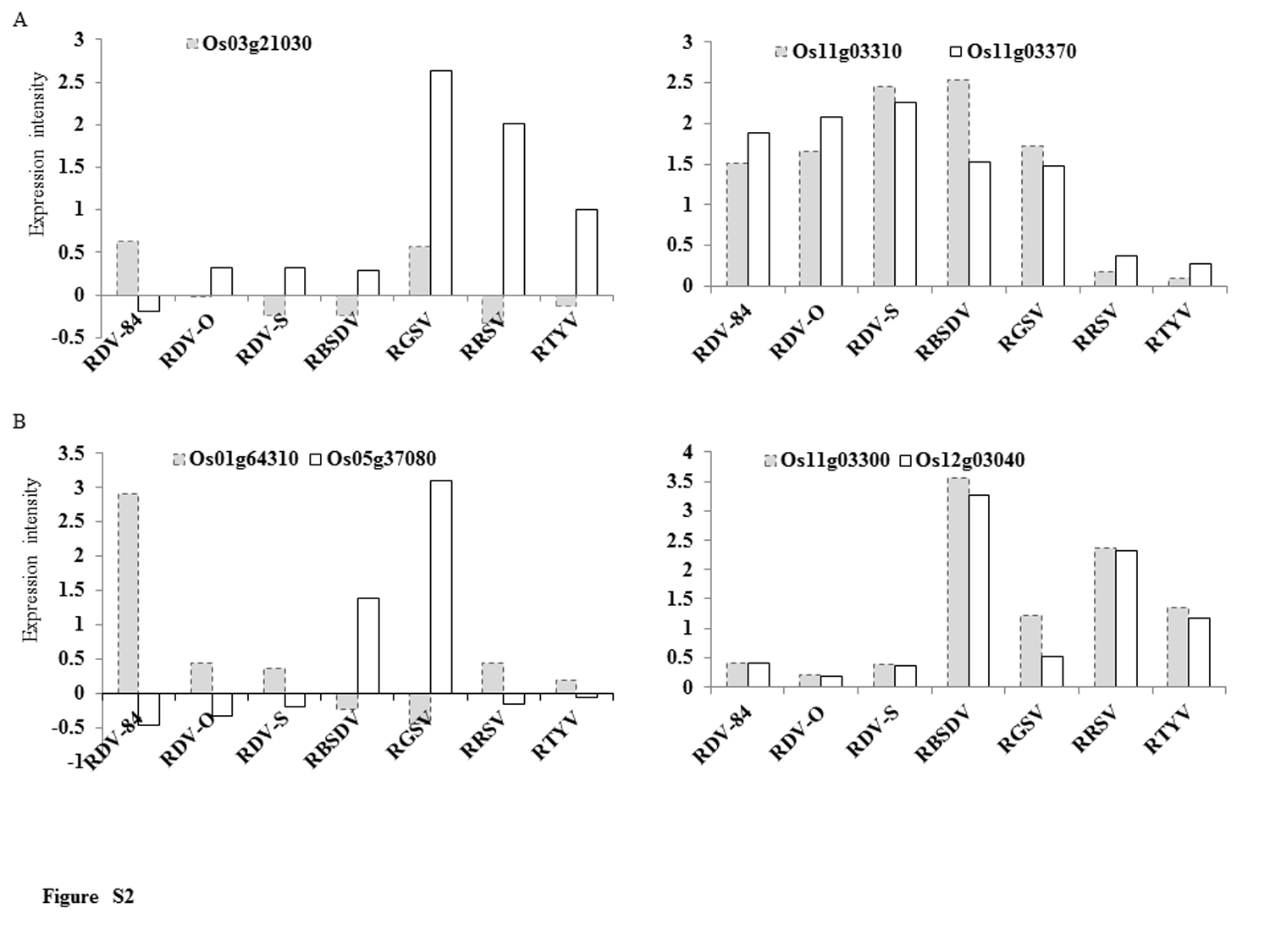

Supplement: Figure S2 — Examples of expression patterns of OsNAC genes found in duplicated regions of the rice genome. (A) Expression patterns of two pairs of tandemly duplicated OsNAC genes. (B) Expression patterns of two OsNAC genes found in segmentally duplicated regions. The different biotic stresses are shown on the X-axis and the expression intensity is on the Y-axis. Gene names are indicated at the top of each graph. Elaborations of virus infection are in Table 1. [file Image2.TIF]
