# Supplementary material for: NAC transcription factor family genes are differentially expressed in rice during infections with Rice dwarf virus, Rice black-streaked dwarf virus, Rice grassy stunt virus, Rice ragged stunt virus, and Rice transitory yellowing virus
Source: Front Plant Sci. 2015 Sep 9;6:676. doi: 10.3389/fpls.2015.00676 (PMC4563162; doi:10.3389/fpls.2015.00676)
Supplement: Supplementary file 3 [file Table1.PDF]

Table S1. The table shows the number of differentially expressed genes (DEGs) during different virus infections. Up-regulated/activated and down-regulated/suppressed genes are mentioned in different folds (e.g. >1.5, >2, and >4) in this table during different virus infections.

	DEG	RDV-84	RDV-O	RDV-S	RBSDV	RGSV	RRSV	RTYV
**Up-regulated or Activated**	> 1.5	19	10	16	21	33	18	2
	> 2	14	8	15	17	26	13	0
	> 4	2	2	8	9	11	5	0
**Down-regulated or Suppressed**	> 1.5	5	6	9	14	9	12	3
	> 2	2	3	8	10	5	7	1
	> 4	1	0	4	3	2	0	0
