# Supplementary material for: NAC transcription factor family genes are differentially expressed in rice during infections with Rice dwarf virus, Rice black-streaked dwarf virus, Rice grassy stunt virus, Rice ragged stunt virus, and Rice transitory yellowing virus
Source: Front Plant Sci. 2015 Sep 9;6:676. doi: 10.3389/fpls.2015.00676 (PMC4563162; doi:10.3389/fpls.2015.00676)
Supplement: Supplementary file 5 [file Table3.DOC]

**Table S3.** Putative *cis*-elements enriched in promoters of rice *NAC* genes.

| *Cis*-element name | Sequence | TF/Motif | Stimulus/tissue |
| --- | --- | --- | --- |
| GAGA8HVBKN3 | GAGAGAGAGAGAGAGA |  |  |
| RYREPEATGMGY2 | CATGCAT |  |  |
| RYREPEATLEGUMINBOX | CATGCAY |  | Seed |
| BOXIINTPATPB | ATAGAA | GT-1 | Light |
| RYREPEATVFLEB4 | ACGTGGTC | ABRE | ABA, drought |
| SV40COREENHAN | GTGGWWHG |  |  |
| ARFAT | TGTCTC | ARF | Auxin |
| PYRIMIDINEBOXHVEPB1 | TTTTTTCC |  | GA3 |
| TATABOX4 | TATATAA |  |  |
| POLASIG2 | AATTAAA |  |  |
| GT1CORE | GGTTAA | GT-1 | Light |
| RYREPEATBNNAPA | CATGCA |  | Seed |
| PYRIMIDINEBOXOSRAMY1A | CCTTTT | BPBF | GA3 |
| IBOXCORE | GATAA |  | Light |
| WBBOXPCWRKY1 | TTTGACY | WRKY | SA |
| LTRE1HVBLT49 | CCGAAA |  | Cold |
| [G-box-like](http://www.bioinformatics2.wsu.edu/Osiris/binding_factors/Gboxlike.html) | CACGTG |  |  |
| [POLASIG1](http://www.bioinformatics2.wsu.edu/Osiris/binding_factors/POLASIG1.html) | AATAAA |  |  |
| [TATABOXOSPAL](http://www.bioinformatics2.wsu.edu/Osiris/binding_factors/TATABOXOSPAL.html) | CACATG |  |  |
| [TATAboxII](http://www.bioinformatics2.wsu.edu/Osiris/binding_factors/TATAboxII.html) | TATTAATA |  |  |
| MYBGAHV | TAACAAA | MYB | GA3 |
| MYB1AT | WAACCA |  | Drought |
| [MYCATERD1](http://www.bioinformatics2.wsu.edu/Osiris/binding_factors/MYCATERD1.html) | CATGTG | MYC | Drought |
| MYCATRD22 | CACATG | MYC | ABA, drought |
| TATCCAYMOTIFOSRAMY3D | TATCCAY | TATCCAY motif and G motif | RAmy3D fused to a GUS & Elecrophoretic mobility shift assay |
| ACGTABREMOTIFA2OSEM | ACGTGKC | ABRE | ABA, seed |
| BRRE | GTG TCG |  |  |
| PBF | WAAAGNG |  |  |
| SURE | GAGAC |  |  |
| ABREOSRAB21 | ACGTSSSC | ABRE | ABA |
| CAAT-motifI | CCAATC |  | |  | Light harvesting | | --- | --- | |  |  | |
| SITEIIBOSPCNA | TGGTCCCAC |  | Meristematic tissue-specific expression |
| CAREOSREP1 | CAACTC | REP-1 gene | GARE, GA, & rice seed; |
| ACGTABREMOTIFA2OSEM | ACGTGKC | ACGT- motif A; ABRE gene | ABA, DRE; |
| GARE1OSREP1 | TAACAGA | REP-1 | GARE, GA, & seed |
| TATAboxIII | TATTAATA | TATAboxIII | Light hervesting |
| TATCCAYMOTIFOSRAMY3D | TATCCAY |  | RAmy3D fused to GUS & elecrophoretic mobility |
| P-box | TGTAAAG |  | Binding with storage proteins |
| DOF3 | GANWAAAGC |  | Electrophoretic mobility shift assay |
| GCN4OSGLUB1 | TGAGTCA | GluB-1, GCN4 motif | Required for endosperm-specific expression; |
| BP5OSWX | CAACGTG | MYC; Wx; Waxy; | Electrophoretic mobility shift assay |
