# Supplementary material for: NAC transcription factor family genes are differentially expressed in rice during infections with Rice dwarf virus, Rice black-streaked dwarf virus, Rice grassy stunt virus, Rice ragged stunt virus, and Rice transitory yellowing virus
Source: Front Plant Sci. 2015 Sep 9;6:676. doi: 10.3389/fpls.2015.00676 (PMC4563162; doi:10.3389/fpls.2015.00676)
Supplement: Supplementary file 7 [file Table5.DOC]

**Table S5.** Tandemly duplicated *OsNAC* genes

| **Cluster** | **Pair** | **Gene** | **Subgroup^1^** | **Duplicate** | **Subgroup^2^** |
| --- | --- | --- | --- | --- | --- |
| 1 | 1 | Os01g01430 | SNAC | Os01g01470 | NAM/CUC3 |
| 2 | 2 | Os02g18460 | ONAC2 | Os02g18470 | ONAC2 |
| 3 | 3 | Os03g21030 | NAM/CUC3 | Os03g21060 | SNAC |
| 4 | 4 | Os03g39050 | ONAC2 | Os03g39100 | ONAC2 |
| 5 | 5 | Os03g61249 | ONAC3 | Os03g61319 | ONAC3 |
| 6 | 6 | Os07g09740 | ONAC2 | Os07g09830 | ONAC2 |
|  | 7 | Os07g09830 | ONAC2 | Os07g09860 | ONAC2 |
| 7 | 8 | Os07g48450 | SNAC | Os07g48550 | NAM/CUC3 |
| 8 | 9 | Os09g38000 | TIP | Os09g38010 | TIP |
| 9 | 10 | Os10g25620 | ONAC4 | Os10g25640 | ONAC4 |
| 10 | 11 | Os10g26240 | ONAC3 | Os10g26270 | ONAC4 |
| 11 | 12 | Os10g27360 | ONAC4 | Os10g27390 | ONAC4 |
| 12 | 13 | Os11g03300 | SNAC | Os11g03310 | NAM/CUC3 |
|  | 14 | Os11g03310 | NAM/CUC3 | Os11g03370 | NAM/CUC3 |
| 13 | 15 | Os11g31330 | ONAC1 | Os11g31340 | ONAC1 |
|  | 16 | Os11g31340 | ONAC1 | Os11g31360 | ONAC1 |
|  | 17 | Os11g31360 | ONAC1 | Os11g31380 | ONAC1 |
| 14 | 18 | Os12g03040 | SNAC | Os12g03050 | NAM/CUC3 |

| ^1^Subgroup of the first gene |
| --- |
| ^2^Subgroup of the tandemly duplicated gene |
