# Supplementary material for: NAC transcription factor family genes are differentially expressed in rice during infections with Rice dwarf virus, Rice black-streaked dwarf virus, Rice grassy stunt virus, Rice ragged stunt virus, and Rice transitory yellowing virus
Source: Front Plant Sci. 2015 Sep 9;6:676. doi: 10.3389/fpls.2015.00676 (PMC4563162; doi:10.3389/fpls.2015.00676)
Supplement: Supplementary file 8 [file Table6.DOC]

| **Table S6.** *OsNAC* genes present on duplicated chromosomal segments of rice. | | | | | | | |
| --- | --- | --- | --- | --- | --- | --- | --- |
| **Gene 1** | | | **Gene 2** | | | **BlastP (e-value)** | **Booｔstrap value** |
| Gene name | Chr | Subgroup^1^ | Gene name | Chr | Subgroup^2^ |  |  |
| *Os01g64310* | 1 | ONAC7 | *Os05g37080* | 5 | ONAC7 | 3.60E-74 | 999 |
| *Os01g66120* | 1 | SNAC | *Os05g34830* | 5 | SNAC | 4.80E-117 | 1000 |
| *Os03g21060* | 3 | SANC | *Os07g48450* | 7 | SANC | 2.00E-92 | 1000 |
| *Os03g56580* | 3 | ANAC34 | *Os07g04560* | 7 | ANAC34 | 1.70E-89 | 1000 |
| *Os03g60080* | 3 | SANC | *Os07g12340* | 7 | SANC | 4.70E-95 | 997 |
| *Os04g59470* | 4 | SND | *Os08g01330* | 8 | SND | 6.00E-55 | 982 |
| *Os08g42400* | 8 | ONAC1 | *Os09g33490* | 9 | ONAC1 | 9.50E-41 | 999 |
| *Os11g03300* | 11 | SNAC | *Os12g03040* | 12 | SNAC | 2.80E-199 | 1000 |
| *Os11g05614* | 11 | ONAC7 | *Os12g05990* | 12 | ONAC7 | 2.80E-89 | 1000 |

| ^1^Subgroup of the first gene |
| --- |
| ^2^Subgroup of the segmentaly duplicated gene |
