# Supplementary material for: NAC transcription factor family genes are differentially expressed in rice during infections with Rice dwarf virus, Rice black-streaked dwarf virus, Rice grassy stunt virus, Rice ragged stunt virus, and Rice transitory yellowing virus
Source: Front Plant Sci. 2015 Sep 9;6:676. doi: 10.3389/fpls.2015.00676 (PMC4563162; doi:10.3389/fpls.2015.00676)
Supplement: Supplementary file 9 [file Table7.DOC]

**Table S7.** Function of NAC transcription factors in virus/pathogen infections.

| Genes/ target genes | Functions | Method | Species | References |
| --- | --- | --- | --- | --- |
| *rim1-1* | Resistance to rice dwarf virus (RDV), susceptible to *rice* transitory yellowing virus (RTYV) and RSV | Knockout | *O. sativa* | Yoshii et al., 2009 |
| *Os02g34970*, *Os02g38130*, *Os11g03310*, *Os11g03370*, *Os11g05614*, *Os12g03050* | RSV, RTSV infections | Microarray | *O. sativa* | Nuruzzaman et al., 2010 |
| *HvNAC6* | *HvNAC6* positively regulates penetration resistant towards *Bl. gramini f.sp. hordei* (*Bgh*) attack | Knockdown/ overexpression | *H*. *vulgare* | Jensen et al., 2007 |
| *ataf1-1* | Loss-of-function mutants have attenuated penetration resistance towards *Bgh* attack | Knockout | *A*. *thaliana* (*At*) | Jensen et al., 2008 |
| *ATAF1, PR1* | ATAF1 negatively regulates  resistance to *B*. *cinerea* | Overexpression/ *ataf1*-*1* and *ataf1-2*, knockout | *A*. *thaliana* | Wu et al., 2009 |
| *ATAF1*, *PR-1, PR-5, NPR1, PDF1.2* | ATAF1 negatively regulates resistance to *P. syringae*, *B*. *cinerea*, *A*. *brassicicola* | Overexpression/ *ataf1-2*, knockout | *A*. *thaliana* | Wang et al., 2009a |
| *ATAF2 , PR1, PR2, PR4, PR5, PDF1.1, PDF1.2* | ATAF2 negatively regulates  resistance to *F*. *oxysporum*,  represses pathogenesis-related  proteins | Overexpression/ knockout | *A*. *thaliana* | Delessert et al., 2005 |
| *ATAF2, PR1, PR2, PDF1.2* | OX = Reduced tobacco mosaic  virus accumulation, increased  pathogenesis-related genes | Overexpression/ knockout | *A*. *thaliana* | Wang et al., 2009a |
| *ATAF2*, NIT2 | Defense hormones, pathogen infection | Overexpression/ knockout | *A*. *thaliana* | Huh et al., 2012 |
| *ANAC019, ANAC055* | Defense disease, JA pathway | Overexpression | *A*. *thaliana* | Bu et al., 2008 |
| *NTL6, PR1, PR2, PR5* | Positive regulator of pathogen  resistance against *P*. *syringae* | Gene silencing/  overexpression | *A*. *thaliana* | Seo et al., 2010 |
| *ANAC042, P450* | Regulation of camalexin biosynthesis, pathogen infection | β- Glucuronidase (GUS)-reporter assays | *A*. *thaliana* | Saga,2012 |
| *SlNAC1* | Increased tomato leaf curl virus  (TLCV) DNA accumulation | Transient  overexpression | *N*. *benthamiana* | Selth et al., 2005 |
| *OsNAC4* | Inducer of HR cell death upon  *Acidovorax avenae* infection, loss of plasma membrane integrity, nuclear DNA fragmentation | Overexpression/  knockdown | *Oryza* (O) *sativa* | Kaneda et al., 2009 |
| *OsNAC6 , PR protein 1, Probenazoleinducible proteins (PBZ1s), DUF26- like Ser/Thr protein kinase,Thioredoxin, Peroxidase,Lipoxygenase,* | Slightly increased tolerance to rice blast disease | Overexpression | *O. sativa* | Nakashima et al., 2007 |
| *OsNAC19* | Disease resistance | Infection | *O. sativa* | Lin et al., 2007 |
| *GRAB1, GRAB2* | Inhibited wheat dwarf virus  replication | Transient  Overexpression | *T*. *monococcum* | Xie et al., 1999 |
| *ATAF2* | Tobacco mosaic virus | Transgenic | Tobaco | Wang et al., 2009b |
| *ONAC122* and *ONAC131*  brome mosaic virus (BMV) | Defense responses against *Magnaporthe grisea* | ----- | *O. sativa* | Sun, 2013 |
| *SlNAC1* | Upregulated during pseudomonas infection | Pathogen infection | *S*.*lycopersicum* | Huang et al., 2012 |
| *CaNAC1* | Defense responses against pathogen | Infection | *C*. *arietinum* | Oh et al., 2005 |
| *GmNAC6* | Responses to biotic signals, osmotic stress-induced | Transctiption | *G*. *max* | [Faria](http://www.ncbi.nlm.nih.gov/pubmed?term=Faria JA%5BAuthor%5D&cauthor=true&cauthor_uid=21943253), 2011 |
| *TLCV*, *SlNAC1* | Enhances viral replication | Overexpression | *L. esculentum* | Selth et al., 2005 |
| *BnNAC14*, *BnNAC485*, *ATAF1* or *ATAF2* | Response to biotic and abiotic stresses including wounding | cDNA libraries | ----- | Hegedus et al., 2003 |
| *Stprx2, StNAC* | Wounding and pathogen response | Transcriptome | *S. tuberosum* | Collinge and Boller, 2001 |
| *NT L4* | ROS under abscisic acid, leaf senescence | Transgenic | *A*. *thaliana* | Lee et al., 2012 |
| *NTL9* | Osmotic stress responses, leaf  senescence | Overexpression  */*knocout | *A*. *thaliana* | Yoon et al., 2008 |
| *MtNAC969* | Symbiotic nodule senescence | Overexpresion | *M*. *truncatula* | de Zélicourt et al., 2012 |
| *VNI2* , *OR/RD* | Leaf senescence | Transcription | *A*. *thaliana* | Seo and Park,a 2011 |
| *Os07g37920*, *Wheat GPC* | Senescence | Overexpression/ RNAi | *O. sativa*, [*T*. *aestivum*](http://en.wikipedia.org/wiki/Common_wheat) | [Distelfeld](http://www.ncbi.nlm.nih.gov/pubmed?term=Distelfeld A%5BAuthor%5D&cauthor=true&cauthor_uid=22278768) et al., 2012 |
| *AtNAP* | Leaf senescence | Overexpression/ RNAi | *A*. *thaliana* | Guo and Gan, 2006 |

**References**

Bu, Q., Jian, H., Li, C.B., Zhai, Q., Zhang, J., Wu, X., [Sun, J](http://www.ncbi.nlm.nih.gov/pubmed?term=Sun J%5BAuthor%5D&cauthor=true&cauthor_uid=18427573)., [Xie, Q](http://www.ncbi.nlm.nih.gov/pubmed?term=Xie Q%5BAuthor%5D&cauthor=true&cauthor_uid=18427573)., and [Li, C](http://www.ncbi.nlm.nih.gov/pubmed?term=Li C%5BAuthor%5D&cauthor=true&cauthor_uid=18427573). (2008). Role of the *Arabidopsis* *thaliana* NAC transcription factors ANAC019 and ANAC055 in regulating jasmonic acid signaled defense responses. *Cell Res*. 18, 756–767.

Collinge, M. and Boller, T. (2001). Differential induction of two potato genes, *Stprx2* and *StNAC*, in response to infection by *Phytophthora infestans* and to wounding. *Plant Mol*. *Biol*. 46, 521–529.

# [**de Zélicourt, A**](http://www.ncbi.nlm.nih.gov/pubmed?term=de Zélicourt A%5BAuthor%5D&cauthor=true&cauthor_uid=22098255)., [**Diet, A**](http://www.ncbi.nlm.nih.gov/pubmed?term=Diet A%5BAuthor%5D&cauthor=true&cauthor_uid=22098255)., [**Marion, J**](http://www.ncbi.nlm.nih.gov/pubmed?term=Marion J%5BAuthor%5D&cauthor=true&cauthor_uid=22098255)., [**Laffont, C**](http://www.ncbi.nlm.nih.gov/pubmed?term=Laffont C%5BAuthor%5D&cauthor=true&cauthor_uid=22098255)., [**Ariel, F**](http://www.ncbi.nlm.nih.gov/pubmed?term=Ariel F%5BAuthor%5D&cauthor=true&cauthor_uid=22098255)., [**Moison, M**](http://www.ncbi.nlm.nih.gov/pubmed?term=Moison M%5BAuthor%5D&cauthor=true&cauthor_uid=22098255)., [**Zahaf, O**](http://www.ncbi.nlm.nih.gov/pubmed?term=Zahaf O%5BAuthor%5D&cauthor=true&cauthor_uid=22098255)., [**Crespi, M**](http://www.ncbi.nlm.nih.gov/pubmed?term=Crespi M%5BAuthor%5D&cauthor=true&cauthor_uid=22098255)., [**Gruber, V**](http://www.ncbi.nlm.nih.gov/pubmed?term=Gruber V%5BAuthor%5D&cauthor=true&cauthor_uid=22098255).,**and** [**Frugier, F**](http://www.ncbi.nlm.nih.gov/pubmed?term=Frugier F%5BAuthor%5D&cauthor=true&cauthor_uid=22098255). (2012). Dual involvement of a Medicago truncatula NAC transcription factor in root abiotic stress response and symbiotic nodule senescence. [***Plant J*.**](http://www.ncbi.nlm.nih.gov/pubmed/?term=The+Plant+Journal+(2012)+70%2C+220–230)**70,** 220-30.

[Delessert, C](http://www.ncbi.nlm.nih.gov/pubmed?term=Delessert C%5BAuthor%5D&cauthor=true&cauthor_uid=16115070)., [Kazan, K](http://www.ncbi.nlm.nih.gov/pubmed?term=Kazan K%5BAuthor%5D&cauthor=true&cauthor_uid=16115070)., [Wilson, I. W](http://www.ncbi.nlm.nih.gov/pubmed?term=Wilson IW%5BAuthor%5D&cauthor=true&cauthor_uid=16115070)., [Van Der Straeten, D](http://www.ncbi.nlm.nih.gov/pubmed?term=Van Der Straeten D%5BAuthor%5D&cauthor=true&cauthor_uid=16115070)., [Manners, J](http://www.ncbi.nlm.nih.gov/pubmed?term=Manners J%5BAuthor%5D&cauthor=true&cauthor_uid=16115070)., [Dennis, E. S](http://www.ncbi.nlm.nih.gov/pubmed?term=Dennis ES%5BAuthor%5D&cauthor=true&cauthor_uid=16115070)., and [Dolferus, R](http://www.ncbi.nlm.nih.gov/pubmed?term=Dolferus R%5BAuthor%5D&cauthor=true&cauthor_uid=16115070). (2005). The transcriptionfactor ATAF2 represses the expression of pathogenesis-related genes in Arabidopsis. *Plant J*. 43, 745–757

[Distelfeld, A](http://www.ncbi.nlm.nih.gov/pubmed?term=Distelfeld A%5BAuthor%5D&cauthor=true&cauthor_uid=22278768)., [Pearce, S. P](http://www.ncbi.nlm.nih.gov/pubmed?term=Pearce SP%5BAuthor%5D&cauthor=true&cauthor_uid=22278768)., [Avni, R](http://www.ncbi.nlm.nih.gov/pubmed?term=Avni R%5BAuthor%5D&cauthor=true&cauthor_uid=22278768)., [Scherer, B](http://www.ncbi.nlm.nih.gov/pubmed?term=Scherer B%5BAuthor%5D&cauthor=true&cauthor_uid=22278768)., [Uauy, C](http://www.ncbi.nlm.nih.gov/pubmed?term=Uauy C%5BAuthor%5D&cauthor=true&cauthor_uid=22278768)., [Piston, F](http://www.ncbi.nlm.nih.gov/pubmed?term=Piston F%5BAuthor%5D&cauthor=true&cauthor_uid=22278768)., [Slade, A](http://www.ncbi.nlm.nih.gov/pubmed?term=Slade A%5BAuthor%5D&cauthor=true&cauthor_uid=22278768)., [Zhao, R](http://www.ncbi.nlm.nih.gov/pubmed?term=Zhao R%5BAuthor%5D&cauthor=true&cauthor_uid=22278768), and [Dubcovsky, J](http://www.ncbi.nlm.nih.gov/pubmed?term=Dubcovsky J%5BAuthor%5D&cauthor=true&cauthor_uid=22278768). (2012). Divergent functions of orthologous NAC transcription factors in wheat and rice. [*Plant Mol*. *Biol*.](http://www.ncbi.nlm.nih.gov/pubmed/?term=Os07g37920%2C+Wheat+GPC) 78, 515–24.

Faria, J. A., Reis, P. A., Reis, M. T., Rosado, G. L., Pinheiro, G. L., Mendes, G. C., and Fontes, E. P. (2011). The NAC domain-containing protein, GmNAC6, is a downstream component of the ER stress- and osmotic stress-induced NRP-mediated cell-death signaling pathway. *BMC Plant Biol*. 11, 129.

[Guo, Y](http://www.ncbi.nlm.nih.gov/pubmed?term=Guo Y%5BAuthor%5D&cauthor=true&cauthor_uid=16640597)., and [Gan, S](http://www.ncbi.nlm.nih.gov/pubmed?term=Gan S%5BAuthor%5D&cauthor=true&cauthor_uid=16640597). (2006). AtNAP, a NAC family transcription factor, has an important role in leaf senescence. [*Plant J*.](http://www.ncbi.nlm.nih.gov/pubmed/16640597) 46, 601–12.

Hegedus, D., Yu, M., Baldwin, D., Gruber, M., Sharpe, A., Parkin, I., [Whitwill, S](http://www.ncbi.nlm.nih.gov/pubmed?term=Whitwill S%5BAuthor%5D&cauthor=true&cauthor_uid=14750526)., and [Lydiate, D](http://www.ncbi.nlm.nih.gov/pubmed?term=Lydiate D%5BAuthor%5D&cauthor=true&cauthor_uid=14750526). (2003). Molecular characterization of *Brassica napus* NAC domain transcriptional activators induced in response to biotic and abiotic stress. *Plant Mol*. *Biol*. 53, 383–397.

[Huang, H](http://www.ncbi.nlm.nih.gov/pubmed?term=Huang H%5BAuthor%5D&cauthor=true&cauthor_uid=22794915)., [Wang, Y](http://www.ncbi.nlm.nih.gov/pubmed?term=Wang Y%5BAuthor%5D&cauthor=true&cauthor_uid=22794915)., [Wang, S](http://www.ncbi.nlm.nih.gov/pubmed?term=Wang S%5BAuthor%5D&cauthor=true&cauthor_uid=22794915)., [Wu, X](http://www.ncbi.nlm.nih.gov/pubmed?term=Wu X%5BAuthor%5D&cauthor=true&cauthor_uid=22794915)., [Yang, K](http://www.ncbi.nlm.nih.gov/pubmed?term=Yang K%5BAuthor%5D&cauthor=true&cauthor_uid=22794915)., [Niu, Y](http://www.ncbi.nlm.nih.gov/pubmed?term=Niu Y%5BAuthor%5D&cauthor=true&cauthor_uid=22794915)., and Dai, S. (2012). Transcriptome-wide survey and expression analysis of stress-responsive NAC genes in *Chrysanthemum lavandulifolium*. [*Plant Sci*.](http://www.ncbi.nlm.nih.gov/pubmed/?term=ClNAC)194**,** 18–27.

[Huh, S. U](http://www.ncbi.nlm.nih.gov/pubmed?term=Huh SU%5BAuthor%5D&cauthor=true&cauthor_uid=22965747)., [Lee, S. B](http://www.ncbi.nlm.nih.gov/pubmed?term=Lee SB%5BAuthor%5D&cauthor=true&cauthor_uid=22965747)., [Kim, H. H](http://www.ncbi.nlm.nih.gov/pubmed?term=Kim HH%5BAuthor%5D&cauthor=true&cauthor_uid=22965747)., and [Paek, K. H](http://www.ncbi.nlm.nih.gov/pubmed?term=Paek KH%5BAuthor%5D&cauthor=true&cauthor_uid=22965747). (2012). ATAF2, a NAC transcription factor, binds to the promoter and regulates *NIT2* gene expression involved in auxin biosynthesis. [*Mol. Cells*](http://www.ncbi.nlm.nih.gov/pubmed/?term=ATAF2%2C+NIT2) 34, 305–13.

Jensen, M. K., [Hagedorn, P. H](http://www.ncbi.nlm.nih.gov/pubmed?term=Hagedorn PH%5BAuthor%5D&cauthor=true&cauthor_uid=18694460)., [de Torres-Zabala, M](http://www.ncbi.nlm.nih.gov/pubmed?term=de Torres-Zabala M%5BAuthor%5D&cauthor=true&cauthor_uid=18694460)., [Grant, M. R](http://www.ncbi.nlm.nih.gov/pubmed?term=Grant MR%5BAuthor%5D&cauthor=true&cauthor_uid=18694460)., [Rung, J. H](http://www.ncbi.nlm.nih.gov/pubmed?term=Rung JH%5BAuthor%5D&cauthor=true&cauthor_uid=18694460)., [Collinge, D. B](http://www.ncbi.nlm.nih.gov/pubmed?term=Collinge DB%5BAuthor%5D&cauthor=true&cauthor_uid=18694460)., and [Lyngkjaer, M. F](http://www.ncbi.nlm.nih.gov/pubmed?term=Lyngkjaer MF%5BAuthor%5D&cauthor=true&cauthor_uid=18694460). (2008). Transcriptional regulation by an NAC (NAMATAF1,2-CUC2) transcription factor attenuates ABA signaling for efﬁcient basal defence towards *Blumeria graminis* f sp *hordei* in Arabidopsis. *Plant J*. 56, 867–880.

Jensen, M. K., [Rung, J. H](http://www.ncbi.nlm.nih.gov/pubmed?term=Rung JH%5BAuthor%5D&cauthor=true&cauthor_uid=17619150)., [Gregersen, P. L](http://www.ncbi.nlm.nih.gov/pubmed?term=Gregersen PL%5BAuthor%5D&cauthor=true&cauthor_uid=17619150)., [Gjetting, T](http://www.ncbi.nlm.nih.gov/pubmed?term=Gjetting T%5BAuthor%5D&cauthor=true&cauthor_uid=17619150)., [Fuglsang, A. T](http://www.ncbi.nlm.nih.gov/pubmed?term=Fuglsang AT%5BAuthor%5D&cauthor=true&cauthor_uid=17619150)., [Hansen, M](http://www.ncbi.nlm.nih.gov/pubmed?term=Hansen M%5BAuthor%5D&cauthor=true&cauthor_uid=17619150)., [Joehnk, N](http://www.ncbi.nlm.nih.gov/pubmed?term=Joehnk N%5BAuthor%5D&cauthor=true&cauthor_uid=17619150)., [Lyngkjaer, M. F](http://www.ncbi.nlm.nih.gov/pubmed?term=Lyngkjaer MF%5BAuthor%5D&cauthor=true&cauthor_uid=17619150)., and [Collinge, D. B](http://www.ncbi.nlm.nih.gov/pubmed?term=Collinge DB%5BAuthor%5D&cauthor=true&cauthor_uid=17619150). (2007). The HvNAC6 transcription factor: a positive regulator of penetration resistance in barley and Arabidopsis. *Plant Mol*. *Biol*. 65, 137–150.

Kaneda, T., Taga, Y., Takai, R., Iwano, M., Matsui, H., Takayama, S. [Isogai, A](http://www.ncbi.nlm.nih.gov/pubmed?term=Isogai A%5BAuthor%5D&cauthor=true&cauthor_uid=19229294)., and [Che, FS](http://www.ncbi.nlm.nih.gov/pubmed?term=Che FS%5BAuthor%5D&cauthor=true&cauthor_uid=19229294). (2009). The transcription factor OsNAC4 is a key positive regulator of plant hypersensitive cell death. *EMBO J*, 28, 926–936.

Lee, S., Seo, P. J., Lee, H. J., and Park, C. M. (2012). [A NAC transcription factor NTL4 promotes reactive oxygen species production during drought-induced leaf senescence in Arabidopsis.](http://www.ncbi.nlm.nih.gov/pubmed/22313226) *Plant J*. 70, 831–44.

Lin, R., Zhaom, W., Mengm, X., Wang, M., and Peng, Y. (2007). Rice gene *OsNAC19* encodes a novel NAC-domain transcription factor and responds to infection by *Magnaporthe grisea*. *Plant Sci*. 172, 120–130.

Nakashima, K., Tran, L. S., VanNguyen, D., Fujita, M., Maruyama, K., [Todaka, D](http://www.ncbi.nlm.nih.gov/pubmed?term=Todaka D%5BAuthor%5D&cauthor=true&cauthor_uid=17587305).  [Ito, Y](http://www.ncbi.nlm.nih.gov/pubmed?term=Ito Y%5BAuthor%5D&cauthor=true&cauthor_uid=17587305)., [Hayashi, N](http://www.ncbi.nlm.nih.gov/pubmed?term=Hayashi N%5BAuthor%5D&cauthor=true&cauthor_uid=17587305)., [Shinozaki, K](http://www.ncbi.nlm.nih.gov/pubmed?term=Shinozaki K%5BAuthor%5D&cauthor=true&cauthor_uid=17587305)., and [Yamaguchi-Shinozaki, K](http://www.ncbi.nlm.nih.gov/pubmed?term=Yamaguchi-Shinozaki K%5BAuthor%5D&cauthor=true&cauthor_uid=17587305). (2007). Functional analysis of a NAC-type transcription factor OsNAC6 involved in abiotic and biotic stress-responsive gene expression in rice. *Plant J*. 51, 617–630.

[Nuruzzaman, M](http://www.ncbi.nlm.nih.gov/pubmed?term=Nuruzzaman M%5BAuthor%5D&cauthor=true&cauthor_uid=20600702)., [Manimekalai. R](http://www.ncbi.nlm.nih.gov/pubmed?term=Manimekalai R%5BAuthor%5D&cauthor=true&cauthor_uid=20600702)., [Sharoni, A. M](http://www.ncbi.nlm.nih.gov/pubmed?term=Sharoni AM%5BAuthor%5D&cauthor=true&cauthor_uid=20600702)., [Satoh, K](http://www.ncbi.nlm.nih.gov/pubmed?term=Satoh K%5BAuthor%5D&cauthor=true&cauthor_uid=20600702)., [Kondoh, H](http://www.ncbi.nlm.nih.gov/pubmed?term=Kondoh H%5BAuthor%5D&cauthor=true&cauthor_uid=20600702)., [Ooka, H](http://www.ncbi.nlm.nih.gov/pubmed?term=Ooka H%5BAuthor%5D&cauthor=true&cauthor_uid=20600702)., and [Kikuchi, S](http://www.ncbi.nlm.nih.gov/pubmed?term=Kikuchi S%5BAuthor%5D&cauthor=true&cauthor_uid=22526427). (2010). Genome-wide analysis of NAC transcription factor family in rice. [*Gene*](http://www.ncbi.nlm.nih.gov/pubmed/?term=Gene%2C+465%3A+30–44)  465, 30–44.

# [**Oh, S. K**](http://www.ncbi.nlm.nih.gov/pubmed?term="Oh SK"%5BAuthor%5D)., [**Lee, S**](http://www.ncbi.nlm.nih.gov/pubmed?term="Lee S"%5BAuthor%5D)., [**Yu, S. H**](http://www.ncbi.nlm.nih.gov/pubmed?term="Yu SH"%5BAuthor%5D)., and [**Choi, D**](http://www.ncbi.nlm.nih.gov/pubmed?term="Choi D"%5BAuthor%5D). (2005). Expression of a novel NAC domain-containing transcription factor (CaNAC1) is preferentially associated with incompatible interactions between chili pepper and pathogens. *Planta* 222, 876–887.

Saga, H., Ogawa, T., Kai, K., Suzuki, H., Ogata, Y., Sakurai, N., Shibata, D., and Ohta, D. (2012). Identification and characterization of ANAC042, a transcription factor family gene involved in the regulation of camalexin biosynthesis in Arabidopsis. *Mol*. *Plant Microbe*. *Interact*. 25, 684–96.

Selth, L. A., [Dogra, S. C](http://www.ncbi.nlm.nih.gov/pubmed?term=Dogra SC%5BAuthor%5D&cauthor=true&cauthor_uid=15608335)., [Rasheed, M. S](http://www.ncbi.nlm.nih.gov/pubmed?term=Rasheed MS%5BAuthor%5D&cauthor=true&cauthor_uid=15608335)., [Healy, H](http://www.ncbi.nlm.nih.gov/pubmed?term=Healy H%5BAuthor%5D&cauthor=true&cauthor_uid=15608335)., [Randles, J. W](http://www.ncbi.nlm.nih.gov/pubmed?term=Randles JW%5BAuthor%5D&cauthor=true&cauthor_uid=15608335)., and [Rezaian, M. A](http://www.ncbi.nlm.nih.gov/pubmed?term=Rezaian MA%5BAuthor%5D&cauthor=true&cauthor_uid=15608335). (2005) A NAC domain protein interacts with *Tomato leaf curl virus* replication accessory protein and enhances viral replication. *Plant Cell* 17, 311–325.

Seo, P. J., and Park, C. M. (2011). Signaling linkage between environmental stress resistance and leaf senescence in Arabidopsis. *Plant Signal Behav*. 6, 1564–6.

[Sun, L](http://www.ncbi.nlm.nih.gov/pubmed?term=Sun L%5BAuthor%5D&cauthor=true&cauthor_uid=23103994)., [Zhang, H](http://www.ncbi.nlm.nih.gov/pubmed?term=Zhang H%5BAuthor%5D&cauthor=true&cauthor_uid=23103994)., [Li, D](http://www.ncbi.nlm.nih.gov/pubmed?term=Li D%5BAuthor%5D&cauthor=true&cauthor_uid=23103994)., [Huang, L](http://www.ncbi.nlm.nih.gov/pubmed?term=Huang L%5BAuthor%5D&cauthor=true&cauthor_uid=23103994)., [Hong, Y](http://www.ncbi.nlm.nih.gov/pubmed?term=Hong Y%5BAuthor%5D&cauthor=true&cauthor_uid=23103994)., [Ding, X.S](http://www.ncbi.nlm.nih.gov/pubmed?term=Ding XS%5BAuthor%5D&cauthor=true&cauthor_uid=23103994)., [Nelson, R.S](http://www.ncbi.nlm.nih.gov/pubmed?term=Nelson RS%5BAuthor%5D&cauthor=true&cauthor_uid=23103994)., [Zhou, X](http://www.ncbi.nlm.nih.gov/pubmed?term=Zhou X%5BAuthor%5D&cauthor=true&cauthor_uid=23103994). and [Song, F](http://www.ncbi.nlm.nih.gov/pubmed?term=Song F%5BAuthor%5D&cauthor=true&cauthor_uid=23103994). (2013). Functions of rice NAC transcriptional factors, ONAC122 and ONAC131, in defense responses against *Magnaporthe grisea*. *Plant Mol*. *Biol*. 81, 41–56.

[Wang, X](http://www.ncbi.nlm.nih.gov/pubmed?term=Wang X%5BAuthor%5D&cauthor=true&cauthor_uid=19737096)., [Basnayake, B. M](http://www.ncbi.nlm.nih.gov/pubmed?term=Basnayake BM%5BAuthor%5D&cauthor=true&cauthor_uid=19737096)., [Zhang, H](http://www.ncbi.nlm.nih.gov/pubmed?term=Zhang H%5BAuthor%5D&cauthor=true&cauthor_uid=19737096)., [Li, G](http://www.ncbi.nlm.nih.gov/pubmed?term=Li G%5BAuthor%5D&cauthor=true&cauthor_uid=19737096)., [Li, W](http://www.ncbi.nlm.nih.gov/pubmed?term=Li W%5BAuthor%5D&cauthor=true&cauthor_uid=19737096)., [Virk, N](http://www.ncbi.nlm.nih.gov/pubmed?term=Virk N%5BAuthor%5D&cauthor=true&cauthor_uid=19737096). [Mengiste, T](http://www.ncbi.nlm.nih.gov/pubmed?term=Mengiste T%5BAuthor%5D&cauthor=true&cauthor_uid=19737096)., and [Song, F](http://www.ncbi.nlm.nih.gov/pubmed?term=Song F%5BAuthor%5D&cauthor=true&cauthor_uid=19737096). (2009a). The Arabidopsis ATAF1, a NAC transcription factor, is a negative regulator of defense responses against necrotrophic fungal and bacterial pathogens. *Mol*. *Plant Microbe Interact*. 22, 1227–1238.

[Wang, X](http://www.ncbi.nlm.nih.gov/pubmed?term=Wang X%5BAuthor%5D&cauthor=true&cauthor_uid=19625399)., [Goregaoker, S. P](http://www.ncbi.nlm.nih.gov/pubmed?term=Goregaoker SP%5BAuthor%5D&cauthor=true&cauthor_uid=19625399)., and [Culver, J. N](http://www.ncbi.nlm.nih.gov/pubmed?term=Culver JN%5BAuthor%5D&cauthor=true&cauthor_uid=19625399). (2009b). Interaction of the *Tobacco mosaic virus* replicase protein with a NAC domain transcription factor is associated with the suppression of systemic host defenses. *J*. *Virol*. 83, 9720–9730.

Wu, Y., [Deng, Z](http://www.ncbi.nlm.nih.gov/pubmed?term=Deng Z%5BAuthor%5D&cauthor=true&cauthor_uid=19752887)., [Lai, J](http://www.ncbi.nlm.nih.gov/pubmed?term=Lai J%5BAuthor%5D&cauthor=true&cauthor_uid=19752887)., [Zhang, Y](http://www.ncbi.nlm.nih.gov/pubmed?term=Zhang Y%5BAuthor%5D&cauthor=true&cauthor_uid=19752887)., [Yang, C](http://www.ncbi.nlm.nih.gov/pubmed?term=Yang C%5BAuthor%5D&cauthor=true&cauthor_uid=19752887)., [Yin, B](http://www.ncbi.nlm.nih.gov/pubmed?term=Yin B%5BAuthor%5D&cauthor=true&cauthor_uid=19752887). [Zhao, Q](http://www.ncbi.nlm.nih.gov/pubmed?term=Zhao Q%5BAuthor%5D&cauthor=true&cauthor_uid=19752887)., [Zhang, L](http://www.ncbi.nlm.nih.gov/pubmed?term=Zhang L%5BAuthor%5D&cauthor=true&cauthor_uid=19752887)., [Li, Y](http://www.ncbi.nlm.nih.gov/pubmed?term=Li Y%5BAuthor%5D&cauthor=true&cauthor_uid=19752887)., [Yang, C](http://www.ncbi.nlm.nih.gov/pubmed?term=Yang C%5BAuthor%5D&cauthor=true&cauthor_uid=19752887)., and [Xie, Q](http://www.ncbi.nlm.nih.gov/pubmed?term=Xie Q%5BAuthor%5D&cauthor=true&cauthor_uid=19752887). (2009). Dual function of Arabidopsis ATAF1 in abiotic and biotic stress responses. *Cell Res*. 19, 1279–1290.

Xie, Q., Sanz-Burgos, A. P., Guo, H., Garcia, J.A., and Gutierrez, C. (1999). GRAB proteins, novel members of the NAC domain family, isolated by their interaction with a geminivirus protein. *Plant Mol*. *Biol*. 39, 647–656.

[Yoon, H. K](http://www.ncbi.nlm.nih.gov/pubmed?term=Yoon HK%5BAuthor%5D&cauthor=true&cauthor_uid=18443413)., [Kim, S. G](http://www.ncbi.nlm.nih.gov/pubmed?term=Kim SG%5BAuthor%5D&cauthor=true&cauthor_uid=18443413)., [Kim, S. Y](http://www.ncbi.nlm.nih.gov/pubmed?term=Kim SY%5BAuthor%5D&cauthor=true&cauthor_uid=18443413)., and [Park, C. M](http://www.ncbi.nlm.nih.gov/pubmed?term=Park CM%5BAuthor%5D&cauthor=true&cauthor_uid=18443413). (2008). Regulation of leaf senescence by NTL9-mediated osmotic stress signaling in Arabidopsis. [*Mol*. *Cells*](http://www.ncbi.nlm.nih.gov/pubmed/18443413) 25, 438–45.

Yoshii, M., Shimizu, T., Yamazaki, M., Higashi, T., Miyao, A., Hirochika, H., and [Omura, T](http://www.ncbi.nlm.nih.gov/pubmed?term=Omura T%5BAuthor%5D&cauthor=true&cauthor_uid=18980655). (2009). Disruption of a novel gene for a NAC-domain protein in rice confers resistance to rice dwarf virus. *Plant J*. 57, 615–625.
